# Global Predictors of COVID-19 Vaccine Hesitancy: A Systematic Review

**DOI:** 10.3390/vaccines10081349

**Published:** 2022-08-18

**Authors:** Carla Pires

**Affiliations:** Escola de Ciências e Tecnologias da Saúde, CBIOS—Universidade Lusófona’s Research Center for Biosciences and Health Technologies, Campo Grande, 376, 1749-024 Lisbon, Portugal; p5558@ulusofona.pt

**Keywords:** vaccine hesitancy, vaccine acceptance, predictors of vaccine hesitancy, COVID-19 vaccines, multivariate regression models, questionnaire-based studies, national studies, PRISMA, systematic reviews

## Abstract

Background: vaccine hesitancy is defined as a delay in the acceptance or refusal of vaccination, even though immunisation is a determinant in reducing the mortality and morbidity associated with Coronavirus Disease 2019 (COVID-19). Aim: to identify and analyse the predictors of COVID-19 vaccine acceptance and/or hesitancy. Methods: a systematic review according to the Preferred Reporting Items for Systematic Reviews and Meta-Analyses (PRISMA) criteria. Keywords: vaccine and (COVID or SARS) and (acceptance or acceptability or willingness or hesitancy or refusal) and (multivariate or regression) and (questionnaire or survey) and national. Databases/resources: PubMed, DOAJ, SciELO and b-on. Timeframe: March 2020–2022. Inclusion criteria: general population, questionnaire-based, calculation of a multivariate regression model and national studies. Quality assessment: application of the National Heart, Lung, and Blood institute (NHLBI) tool. Results: a total of 37 studies were selected, whose overall rate was fair. The most predominant predictors of vaccine hesitancy were a lower perceived risk of getting infected, a lower level of institutional trust, not being vaccinated against influenza, lower levels of perceived severity of COVID-19, or stronger beliefs that the vaccination would cause side effects or be unsafe. Discussion and conclusion: the identified predictors can be used to design tailored health policies and/or public health interventions, or to evaluate subjects’ vaccine hesitancy.

## 1. Introduction

As of 20 May 2022, there have been 521,920,560 confirmed cases of Coronavirus Disease 2019 (COVID-19), with 6,274,323 deaths, according to the World Health Organization (WHO) COVID-19 dashboard [1]. Globally, only 65.7% of the world population (7.9 billion) and 15.9% of people in low-income countries have, respectively, received at least one dose of a COVID-19 vaccine up to May 2022. For instance, the share of people vaccinated against COVID-19 (at least one dose) was, as follows: United Arab Emirates (99%); China (89%); United States of America (USA) (78%); United Kingdom (78%); and Nigeria (13%) [2,3]. The WHO’s strategy to achieve global COVID-19 vaccination by mid-2022 was only partially achieved, especially in low-income countries. According to this strategy, the following stratified vaccination coverage rates have been previously defined: low (<10%); medium (10–40%); high (41–70%); and very high (>70%) [4].

The COVID-19 pandemic also generated significant macroeconomic losses, such as a reduction in Gross Domestic Product (GDP) and increased microeconomic expenses across multiple countries (e.g., an increase of USD 2082.65 ± 345.04 to USD 2990.76 ± 545.98 for the patients admitted to an Intensive Care Unit (ICU) and negative financial outputs: job loss, the inability to meet financial obligations or essential needs, or using savings to meet financial obligations) [5,6].

COVID-19 vaccines were quickly developed, with various pharmaceutical companies announcing early results of large clinical trials by late November 2020, around nine months after the declaration of a COVID-19 pandemic by the WHO on 11 March 2020 [7,8]. Currently, there are five COVID-19 vaccines authorised in the European Union: Comirnaty (developed by BioNTech and Pfizer); Spikevax (previously COVID-19 Vaccine Moderna); Vaxzevria (previously COVID-19 Vaccine AstraZeneca); Jcovden (previously COVID-19 Vaccine Janssen); and Nuvaxovid [9]. COVID-19 vaccination is essential to mitigating the pandemic, reducing the likelihood of passing the virus on to others, preventing infections in a limited number of cases, saving lives, offering robust protection against serious illness, hospitalisation and death, reducing the number of symptomatic cases, preventing the emergence of variants and reducing the global macro- and microeconomic costs [5,6,10,11,12,13]. Thus, vaccine hesitancy (“a delay in acceptance or refusal of vaccination, despite the availability of vaccination services”) is a global threat [14,15].

Vaccine hesitancy is a complex phenomenon, conditioned by diverse factors, such as complacency (if the perceived risks of vaccine-preventable diseases are low and vaccination is not deemed a necessary preventive action), convenience (availability, affordability and willingness to pay, geographical accessibility, accessibility (ability to understand and the appeal of immunisation services affect uptake) and confidence (trusting the effectiveness and safety of vaccines, health services and healthcare professionals and/or the motivation of policymakers) (3C model) [14]. Alternatively, the 2017 increasing vaccination model is based on five pillars, which also explain subjects’ vaccine hesitancy/acceptance: (i) what people think and feel: perceived risk, worry, confidence, trust and safety concerns; (ii) social processes: provider recommendation, social norms, gender norms, equity, information sharing and rumours; (iii) motivation: readiness, willingness, intention and hesitancy; (iv) practical issues: vaccine availability, convenience, costs, service quality and satisfaction, requirements, incentives and intervention fatigue; and (v) vaccination: schedule appointment, consent, accept vaccine, delay and refuse [16,17].

Scientists and decision-makers are required to investigate the scale and determinants of vaccine hesitancy in each setting/country, with the aim of developing tailored public health strategies that successfully address the problems of vaccine hesitancy [18]. Diverse predictors of COVID-19 vaccine hesitancy were estimated across diverse regions/countries in the last two years, with females, low education/literate subjects, people who have not previously accepted the influenza vaccine and people who declared that they did not trust in government and health authorities more likely to be vaccine-hesitant [19,20].

Thus, the study aims were (i) to identify and analyse the most relevant global predictors of COVID-19 vaccine acceptance and/or hesitancy, which were specifically estimated through multivariate regression models, (ii) to characterise the most relevant predictors per country/region (e.g., contradictory predictors) and (iii) to compare the findings/conclusions of the present systematic review with those from previous similar/related systematic reviews.

Research question: What are the most predominant predictors of vaccine hesitancy at a global level?

## 2. Materials and Methods

### 2.1. Type of Review and Followed Criteria: PRISMA, PICOS and Inclusion and Exclusion Criteria

A systematic review was carried out. The Preferred Reporting Items for Systematic Reviews and Meta-Analyses (PRISMA) and PICOS criteria (P: participants; I: intervention; C: comparisons; O: outcomes; S: study design) were followed to ensure a rigorous selection and reporting of studies (Table 1) [21,22]. The PRISMA 2020 Checklist and PRISMA 2020 flow diagram can be consulted at http://www.prisma-statement.org/ (accessed on 13 August 2022) [22]. All study findings were double-checked and documented/archived for later consultation.

### 2.2. Followed Methodology per Defined Study Aim

#### 2.2.1. Study Objectives 1 and 2

The study objectives (i) and (ii) were developed based on study findings (Appendix A). All data were collected in a MS Excel file, with quantification of the global occurrences/frequencies of predictors, identification of contradictory predictors and calculation of the predictors per region (subgroup analysis).

#### 2.2.2. Study Objective 3

Two meta-analyses and systematic reviews on the same topic were identified in PubMed, b-on, SciELO, DOAJ and Cochrane Library on 2 May 2022. The keywords and search methodology were related to those applied in the present systematic review (see Section 2.3). The covered timeframe, the main findings and the conclusions of these reviews are presented in Table 2 [19,20].

The present systematic review was not classified as an update to a previous systematic review, based on the following definition: “an update asks a similar question with regard to the participants, intervention, comparisons, and outcomes (PICO) and has similar objectives; thus, it has similar inclusion criteria” [23]. Neither of the two previous similar/related systematic reviews on the same topic used the same inclusion or exclusion criteria as this study (see Table 1 and Table 2) [19,20].

### 2.3. Keywords, Search Strategy, and Timeframe

The selected keywords were as follows: vaccine and (COVID or SARS) and (acceptance or acceptability or willingness or hesitancy or refusal) and (multivariate or regression) and (questionnaire or survey) and national. Singular keywords were used instead of plurals to cover the maximum number of related words (i.e., singulars and plurals). No filters were applied, except for PubMed, where the defined timeframe was previously defined. The keywords were conveniently selected based on the study objectives. For instance, “COVID or SARS” were used as keywords instead of “COVID-19 or SARS-CoV-2”, with the aim of identifying more studies. As recommended, a broad and heterogeneous set of keywords were defined to increase the study precision and accuracy [22].

Particularly, the following stream of keywords: “vaccine and (COVID or SARS) and (acceptance or acceptability or willingness or hesitancy or refusal) and (multivariate or regression) and (questionnaire or survey) and national” was browsed in the main screening tool of each one of the selected databases/resources. Results were rechecked using the individual streams of keywords, such as “COVID and acceptance and multivariate and questionnaire and national” or “COVID and acceptance and regression and questionnaire and national”. All possible combinations of keywords were searched.

The timeframe was defined as being between the beginning of the pandemic (11 March 2020) and March 2022 to identify the highest number of works/papers.

### 2.4. Screened Databases/Resources and Dates of Data Collection

Four databases/resources were screened (PubMed, DOAJ, SciELO and b-on), because they comprise a high number of journals [24,25,26,27]. B-on provides access to thousands of journals through diverse resources, such as Academic Search Complete, Current Contents (ISI) or Web of Science. The full list of b-on contents can be consulted at https://www.b-on.pt/en/collections/ (accessed on 13 August 2022) [27]. SciELO was selected to cover Brazilian, Spanish, Latin American and Portuguese papers [26]. In general, the journals covered in these resources are peer-reviewed.

The dates of data collection per screened database were as follows: PubMed (2 April 2022), DOAJ (2 April 2022), SciELO (2 April 2022) and b-on (17 April 2022) [24,25,26,27].

### 2.5. Data Collection Methodology and Quality Control Assessment

First, all abstracts were read. Studies were immediately excluded if they were not compliant with the inclusion criteria. Second, the preselected papers were downloaded, sequentially numbered and archived. Third, the preselected papers were fully read to recheck their compliance with the inclusion/exclusion criteria. Fourth, data were collected in a tabular format according to Appendix A, i.e., a standardised data extraction form was used.

All data were at least double-checked, considering that only one author carried out the present systematic review. However, errors in the identification and selection of papers were not expected, since the inclusion criteria were very simply and clearly elaborated. After printing, highlighting and reading all papers, the most relevant sections were directly copied and pasted to Appendix A (data collection). These copied and pasted sentences were rephrased, except for the study objectives. Study objectives were integrally copied and pasted, to maintain the original version. Duplicates were manually identified. When applicable, data were transported to an Excel file to compute/calculate descriptive statistics (e.g., sum and average number of participants or frequencies of predictors). The adopted methodological procedures were intended to safeguard the quality and validity of data extraction.

#### Quality Assessment of the Selected Papers

The impact factor from the Journal Citation Reports (JCR) and the quartile according to Scimago Journal & Country Rank of the selected papers are presented in Appendix A [28,29]. Two different metrics were conveniently defined to constitute a robust and diversified indicator of the journals’ quality.

Additionally, a mapping analysis of the most impactful keywords (i.e., related and repeated keywords; the minimum considered number of occurrences of a keyword was two) of the selected papers was carried out to recheck/confirm the main topics covered by the selected papers. A mapping analysis of the authors of the selected papers was also carried out to find possible connections/collaborations between them. Both mapping analyses were carried out with VOSviewer version 1.6.18, which has been developed by Nees Jan van Eck and Ludo Waltman at Leiden University’s Centre for Science and Technology Studies (CWTS) [30]. VOSviewer version 1.6.18 was downloaded and used by the study author to carry out the present mapping analysis in Lisbon, Portugal (July 2022). 

The National Heart, Lung, and Blood institute (NHLBI) quality assessment tool for Observational Cohort and Cross-Sectional Studies was applied to assess/check the methodological quality (risk of bias) of the selected papers [31,32]. This tool was developed by the NHLBI in 2013 and is available for free at https://www.nhlbi.nih.gov/health-topics/study-quality-assessment-tools (accessed on 13 August 2022). The tool was administered in accordance with the instructions displayed on the National Institutes of Health (NIH) website [32] by the study author in Lisbon, Portugal (July 2022); some scoring adaptations were created, as below described. The original questions of the NHLBI quality assessment tool are presented below (three types of replies are possible, yes, no and other, with “other” assuming the following options: CD, cannot determine; NA, not applicable; NR, not reported) [32].

1. Was the research question or objective in this paper clearly stated?
2. Was the study population clearly specified and defined?
3. Was the participation rate of eligible persons at least 50%?
4. Were all the subjects selected or recruited from the same or similar populations (including the same time period)? Were inclusion and exclusion criteria for being in the study prespecified and applied uniformly to all participants?
5. Was a sample size justification, power description or variance and effect estimate provided?
6. For the analyses in this paper, were the exposure(s) of interest measured prior to the outcome(s) being measured? * (No)
7. Was the timeframe sufficient so that one could reasonably expect to see an association between exposure and outcome if it existed? * (No)
8. For exposures that can vary in amount or level, did the study examine different levels of the exposure as it related to the outcome (e.g., categories of exposure, or exposure measured as a continuous variable)? ** (not applicable)
9. Were the exposure measures (independent variables) clearly defined, valid, reliable and implemented consistently across all study participants?
10. Was the exposure(s) assessed more than once over time? (i.e., Was the questionnaire administered at least two times in different moments?/exposure or evaluation of independent variable).
11. Were the outcome measures (dependent variables) clearly defined, valid, reliable and implemented consistently across all study participants?
12. Were the outcome assessors blinded to the exposure status of participants? ** (not applicable)
13. Was loss to follow-up after baseline 20% or less? (i.e., % of participants who did not reply to the second questionnaire; if applicable)
14. Were key potential confounding variables measured and adjusted statistically for their impact on the relationship between exposure(s) and outcome(s)?

According to the instructions of the NIH website, the exposure is the independent variable (i.e., willingness to get a COVID-19 vaccine), and the replies to questions 6 and 7 should be ”no” for cross-sectional studies (*) [32].

Questions 8 (“If the exposure can be defined as a range (examples: drug dosage, amount of physical activity, amount of sodium consumed)”) and 12 (Blinding means that outcome assessors did not know whether the participant was exposed or unexposed) were classified as not applicable to the selected studies (**) and were excluded. Questions 6 and 7 were also excluded, since they are not applicable to cross-sectional studies, and all evaluated studies (n = 37) are cross-sectional.

Scores were previously defined, as follows: 1 for yes; 0 for no; 0.5 for features not reported (i.e., if it is not possible to check if authors have carried out (or not) a certain evaluation/procedure); and 0.5 for the option “cannot determine”, since published data were insufficient to calculate/check a certain feature.

The paired questions were as follows: questions 4.1(Were all the subjects selected or recruited from the same or similar populations (including the same time period)?) and 4.2 (Were inclusion and exclusion criteria for being in the study prespecified and applied uniformly to all participants?), and questions 10 (Was the exposure(s) assessed more than once over time?) and 13 (Was loss to follow-up after baseline 20% or less?); the scoring criteria were: 0.5 for yes; 0 for no; 0.25 for features not reported; and 0.25 for the option “cannot determine”. Questions 4.1 and 4.2 were paired (analysed together) to respect the original version of the tool, and questions 10 and 13 were paired (analysed together), since they are inter-related.

The previously defined rating was good (100% to 90%), fair (less than 90% to 80%) or poor (less than 80%).

### 2.6. Dates of the First Administered Vaccine per Each Studied Country

The dates of the first administered vaccine in each studied country/region can be consulted in Table 3. The presented dates were used to determine if the selected studies were carried out before or after the beginning of COVID-19 vaccination (see Appendix A).

### 2.7. Definitions: Global Predictors of Vaccine Hesitancy, Contradictory Predictors of Vaccine Hesitancy and Most Frequent Predictors of Vaccine Hesitancy per Country or Region

Definitions were conveniently defined to cover the maximum number of predictors of vaccine hesitancy/acceptance:

- Global predictors of vaccine hesitancy: predictors identified in at least two of the selected studies (i.e., frequency of two or more).
- Contradictory predictors of vaccine hesitancy: contrary/opposite variables/predictors, which explain vaccine hesitancy in at least two countries/regions (e.g., females vs. males).
- Most frequent predictors of vaccine hesitancy per country/region: predictors that were present in at least 35% of the selected studies. The selected studies were grouped into nine countries/regions: USA, China, UK, Australia, Asiatic countries, European Union, Latin America and the Caribbean and South Africa (see Appendix A).

## 3. Results

### 3.1. Selected Studies

**Figure 1:**
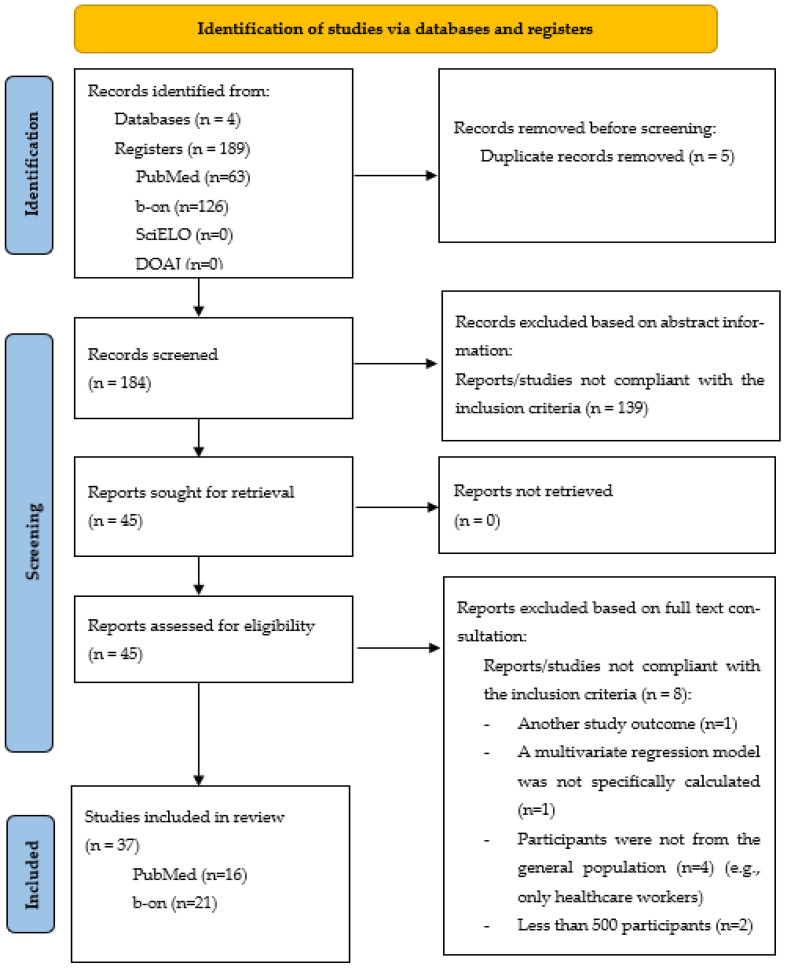
PRISMA 2020 flow diagram for new systematic reviews [55]: studies about the predictors of COVID-19 vaccine hesitancy/acceptance, which were specifically calculated through multivariate regression models.

Globally, 37 papers were selected: 16 PubMed and 21 bon, 0 DOAJ and 0 SciELO papers (Figure 1).

### 3.2. Study Findings

The study findings of each selected study (n = 37) are presented in Appendix A, as follows: studies involving multiple countries (n = 3) [56,57,58]; USA (n = 7) [59,60,61,62,63,64,65]; China (n = 4) [66,67,68,69]; UK (n = 3) [70,71,72]; Australia (n = 3) [73,74,75]; Asia (n = 11) (United Arab Emirates (n = 2); Indonesia (n = 1); Saudi Arabia (n = 1); Jordan (n = 1); Israel (n = 1); Qatar (n = 1); Pakistan (n = 1); Hong Kong (n = 1); Lebanon (n = 1); South Korea (n = 1)) [76,77,78,79,80,81,82,83,84,85,86]; European Union (n = 2) (Italy and Croatia) [87,88]; Latin America (n = 2) (Mexico and Brazil) [89,90]; the Caribbean (n = 1) [91] (Trinidad and Tobago); and Africa (n = 1) (South Africa) [92].

The selected studies were published between 2020 and 2022:2020 (n = 7), 2021 (n = 23) and 2022 (n = 7). The average number of respondents was at least 868,742 (SD = 79,631.5; maximum 459,235; and minimum 615). Logistic regression models were used in 28 (75.7%) out of 37 of the selected studies (Appendix A).

Detailed information on the author, year, geographic region, screened database, paper metrics, study aim, sample size (number of participants that completed the study, i.e., valid respondents), main sociodemographic characteristics, methods, date of administration of questionnaire/survey, main findings and a brief discussion and conclusion are described in Appendix A.

#### 3.2.1. Quality Control Assessment

##### Paper Metrics and Mapping Analysis of the Journals from the Selected Papers

Looking at the journals of the 37 selected papers, only 5 journals did not have an impact factor JCR (Social Sciences, Population Medicine, Cureus and Journal of Pharmaceutical Policy and Practice) [93]. However, all the journals without an impact factor JCR were indexed in at least one of the following resources: PubMed, Web of Science or Scopus. Excluding the five journals without impact factors, the descriptive statistic of the impact factor JCR was: maximum 6.461; minimum = 2.221; average = 4.176; and SD = 1.098). The quartiles according to the Scimago Journal & Country Rank were as follows: Q1 (n = 29); Q2 (n = 4); Q3 (n = 1); and without attribution of a quartile (n = 3).

**Figure 2:**
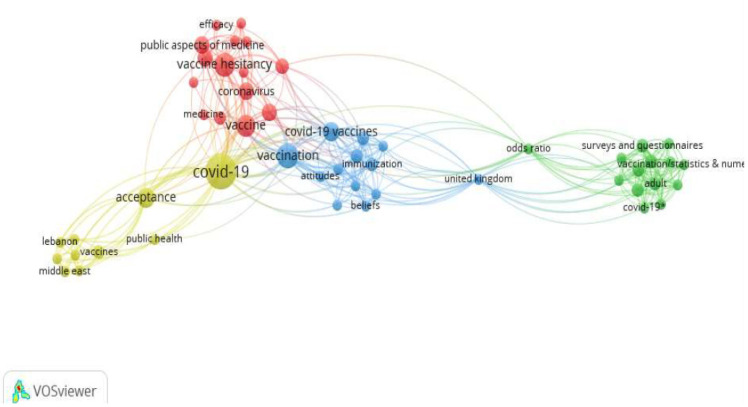
Mapping analysis of keywords from the 37 selected studies.

Regarding the keywords mapping analysis [30], from the 190 identified keywords (n = 37 selected papers), 52 keywords met the threshold (i.e., the minimum considered number of occurrences of a keyword was two) (Figure 2).

**Figure 3:**
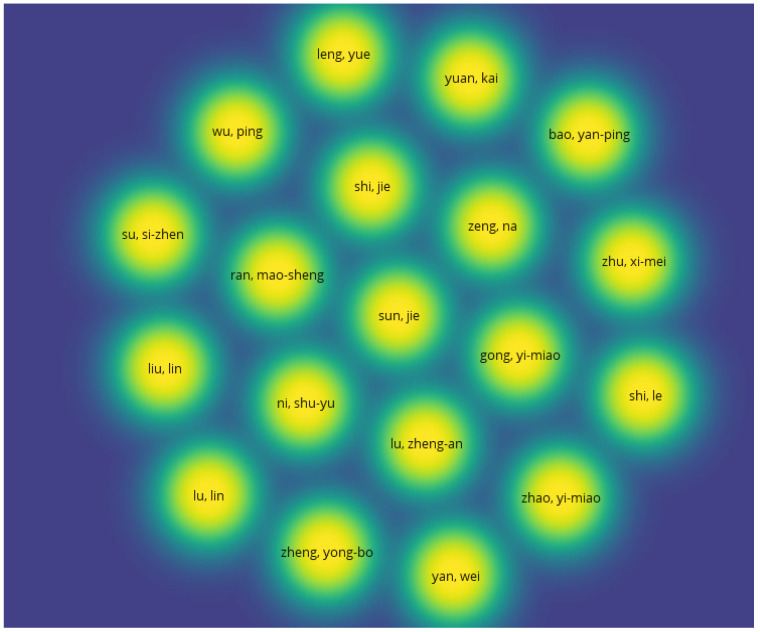
Mapping analysis of the authors from the 37 selected studies.

Overall, 233 authors were involved in the 37 selected papers, of which only 19 authors were connected to each other (Figure 3) (mapping analysis of authors).

##### NHLBI Quality Assessment Tool

The global rating of the 37 selected studies was 82.1% (fair) (273.5 out of 333 points) (Table 4) [32].

It is important to note that excluding two questions—(i) Was the exposure(s) assessed more than once over time? (i.e., Was the questionnaire administered at least two times in different moments?) and (ii) Was loss to follow-up after baseline 20% or less? (e.g., % of participants who did not reply to the second questionnaire)—changed the overall rate of the 37 selected studies to good (91.2%). Additionally, the participation rate was not reported in 20 of the 37 studies, which compromised the evaluation of the item: participation rate of eligible persons (at least 50%).

#### 3.2.2. Studies Carried out before and after the Beginning of the Pandemic

Classification of the 37 selected studies was as follows: no details about the precise implementation date (n = 1) [79]; involving two time periods (i.e., studies carried out before and after the beginning of the COVID-19 vaccination period) (n = 2) [82,92]; studies carried out before the beginning of the COVID-19 vaccination period in the country/region (n = 25) [56,58,59,60,61,62,66,67,68,70,71,73,74,75,76,77,78,80,81,83,84,85,86,87,89]; and studies carried out after the beginning of the COVID-19 vaccination period in the country region (n = 9) [57,63,64,65,69,72,88,90,91] (Appendix A).

In general, COVID-19 vaccination started in December 2020 or January 2021 in developed countries. The exceptions regarding the selected studies were as follows: February 2021 (Japan and South Korea); March 2021 (Lebanon, South Africa and Trinidad and Tobago) and May 2021 (Pakistan) (Table 3). The findings from the 37 selected studies were analysed together, since almost all the selected studies were carried out before the beginning of the COVID-19 vaccination period in the country/region (n = 25), and the remaining studies started a few months after the beginning of the vaccination campaigns. Additionally, data from COVID-19-vaccinated individuals were only collected in one study (Hao et al., 2022) [65]; i.e., vaccine hesitancy was quantified through the intention or likelihood of getting a COVID-19 vaccine in the remaining selected studies. Finally, the question(s) and/or scale(s) used to quantify the intention to get a COVID-19 vaccine were not equal/standardised between the 37 selected studies. Thus, it was not possible to directly compare the findings from the studies carried out before or after the beginning of the COVID-19 vaccination period campaigns, and/or involving both periods.

### 3.3. Previous Similar/Related Systematic Reviews: Commonly Selected Studies

Globally, only 7 (18.9%) of the 37 selected studies were common between the present systematic review and the two previous similar/related systematic reviews [19,20]. The common studies were as follows:

- The study of Reiter et al. (2020) [59] was also selected/included in the two previous similar/related systematic reviews [19,20];
- The studies of Dong et al. (2020) and Harapan et al. (2020) [66,78] were also selected/included in the systematic review of Wang et al. (2021) [19];
- The studies of Al-Mohaithef et al. (2020) [79]; El-Elimat et al. (2021) [80]; Wang et al. (2020) [67]; and Sherman et al. (2021) [71] were also selected/included in the systematic review of Roy et al. (2022) [20].

These findings confirm the relevance of the present work since most of the selected studies were not selected/identified in the two previous similar systematic reviews. The number of selected studies (n = 37) was similar to the number identified/selected studies in the two previous similar/related systematic reviews by Wang et al. (2021) (n = 38) and Roy et al. (2022) (n = 48) [19,20], which supports the validity of the present systematic review.

### 3.4. Global Predictors, Single Occurrences and Contradictory Predictors of Vaccine Hesitancy

#### 3.4.1. Global Predictors of Vaccine Hesitancy

The global predictors of vaccine hesitancy (i.e., predictors identified in at least two studies) are presented in Table 5. The identification of the global predictors of vaccine hesitancy was based on the findings of the multivariate models from the 37 selected studies.

#### 3.4.2. Predictors of Vaccine Hesitancy Identified in Just One Study (Single Occurrences)

Predictors of vaccine hesitancy identified in just one study (2.7% of the 37 selected studies) were as follows: student, retired, age (no additional information), unstable job status, higher income, no religious affiliation, lower socioeconomic status and working in the informal sector, experienced worsening health status during pandemic, not internationally traveling in 2020, less trust in science, declaring to not know about the non-natural origin of the virus, not believing in the seriousness of the COVID-19 situation, less or no fear of COVID-19, infrequent users of traditional media, relying upon social media for virus information/people who report social media as their main source of news, people who use social media for 3 or more hours daily, less positive general COVID-19 vaccination beliefs and attitudes, fewer beliefs about social acceptability of a COVID-19 vaccine, a lower perceived need for vaccination, lower proportion of their family or close friends have already received it, opinion of healthcare provider/if respondents thought their healthcare provider would not recommend vaccination, lower cues to action (triggers for receiving COVID-19 vaccination by four items, including recommendations by the government, physicians, family members and friends, respectively), lower knowledge scores for SARS-CoV-2 infection, assigning importance to the vaccine’s country of origin, living in a certain region, residing in cities, no family member died or admitted to ICU for COVID-19, having already tested positive for COVID-19, not having hypertension, having a food allergy, not being available to pay these vaccines, not being prosocial, the belief that the government restrictions were too lenient, the frequency of socialising prior to the pandemic (goes out frequently), shorter duration of action (less than 12 or 18 months), number of shots (more than one injection), production place (non-imported vaccines) and higher price.

#### 3.4.3. Contradictory Predictors of Vaccine Hesitancy

The contradictory predictors of vaccine hesitancy (i.e., contrary explanatory variables/predictors of vaccine hesitancy/acceptance) are presented next.

##### Females vs. Males

Being female was a predictor of vaccine hesitancy (n = sixteen), as determined by studies from the USA (four studies) [60,61,62,64]; China (two studies) [67,69]; Australia (two studies) [69,70]; Asiatic countries (six studies) [76,77,80,81,82,83]; Croatia (one study) [88]; and Mexico (one study) [89]. Male was a predictor of vaccine hesitancy (n = five), as determined by studies involving multiple countries, including the European Union (three studies) [56,57,58]; Australia (one study) [73]; and Brazil (one study) [90].

##### Younger vs. Older

Being younger was a predictor of vaccine hesitancy (n = thirteen), according to one study involving multiple countries [58] and studies from the USA (three studies) [62,63,65], China (one study) [69], the UK (one study) [71], Australia (one study) [73], Asiatic countries (four studies) [79,80,81,86], Croatia (one study) [88] and South Africa (one study) [92]. Being older was a predictor of vaccine hesitancy (n = four), as found by studies from the United Arab Emirates (two studies) [76,77], Mexico (one study) [89] and Brazil (one study) [90].

##### Minorities vs. Whites or Other Major Ethnic Groups

Minority group was a predictor of vaccine hesitancy (n = eight), as determined by studies from the USA (four studies) [59,62,63,64], the UK (one study) [70], Asiatic countries (two studies) [81,82] and South Africa [92]. Major ethnic group was a predictor of vaccine hesitancy (White or other groups) (n = five), according to studies by the USA (one study) (Hao et al., 2022), Asiatic countries (three studies) [76,77,82] and Brazil (one study) [90].

##### Lower Level of Education vs. Higher Level of Education

A lower level of education was a predictor of vaccine hesitancy (n = seven), as determined by studies from multiple countries (one study) [58], the USA (one study) [60], the UK (one study) [70], Croatia (one study) [88], Latin America (two studies) [89,90] and South Africa (one study) [92]. A higher level of education was a predictor of vaccine hesitancy (n = three), as determined by studies from the European Union (one study) [57]; Australia (one study) [74]; and the United Arab Emirates (Asia) (one study) [76].

##### Lower Income vs. Higher Income

Lower income was a predictor of vaccine hesitancy (n = seven), as determined by studies from the USA (three studies) [60,61,63]; the UK (one study) [70]; South Korea (Asia) (one study) [86]; Brazil (Latin America) (one study); and South Africa (Africa) (one study) [92]. Higher income was a predictor of vaccine hesitancy in one study (China) [69].

##### Not Residing in Cities or Living in Smaller Settlements vs. Residing in Cities

Not residing in cities or living in smaller settlements was a predictor of vaccine hesitancy (n = five), as determined by studies involving multiple countries (two studies) and studies from [57,58] Lebanon (Asia) (one study) [85]; Croatia (European Union) (one study) [88]; and Brazil (Latin America) [90]. Residing in cities was a predictor of vaccine hesitancy in one study (China) [69].

##### Unemployed vs. Employed

Being unemployed was a predictor of vaccine hesitancy (n = three), as determined by studies involving multiple countries (two studies) [57,58] and a study from the United Arab Emirates (Asia) (one study) [76]. Being employed was a predictor of vaccine hesitancy (n = two), as determined by studies from the USA (one study) [60] and Jordan (Asia) (one study) [80].

### 3.5. Most Frequent Predictors of Vaccine Hesitancy per Country or Region

The most frequent predictors of vaccine hesitancy per country or region (i.e., predictors/variables identified in at least 35% of studies) are presented in Table 6. The two studies reporting results from different regions were not included in the analysis of Table 6 [56,58].

## 4. Discussions

Globally, the selected studies covered a broad number of countries/regions (USA, China, UK, Australia, Asiatic countries, European Union, Latin America and the Caribbean and South Africa), and accounted for the participation of at least 868,742 respondents, although around half of the participants were from the USA. Logistic regression models were adopted in almost all studies, supporting an adequate comparison of study results and a precise calculation of variables/predictors. In general, only around half to two-thirds of the respondents of the selected studies declared themselves to be willing to get a COVID-19 vaccine, with higher declaration rates of acceptance in China, Australia, Indonesia and Brazil at >80% [67,68,73,78,90], and lower declaration rates of acceptance in Jordan, Israel, Qatar and Hong Kong at <45% [80,81,82,84], which confirms that vaccine hesitancy remains a global public health and economic problem, implying a direct negative impact on the mitigation of COVID-19 and on the control of mortality and morbidity. The ideal universal goal of COVID-19 vaccine coverage is at least 70%, which is especially relevant for the high-priority groups (e.g., geriatric patients) [4,94].

In general, the journals of the selected papers presented a high impact factor JCR, and the majority were from quartile one (Q1) according to the Scimago Journal & Country Rank, which seems to support the quality, validity and relevance of the selected studies. The quality of the selected papers was also confirmed by application of the NHLBI tool [32]. Additionally, the mapping analysis of keywords carried out with VOSviewer confirmed an inter-relationship between the selected keywords within the scope/aims of the present systematic review, supporting the confirmation of the study validity. In contrast, the mapping analysis of authors carried out with VOSviewer confirmed a limited number of connections (only 19 authors), which seems to indicate the need for more international collaborations, regarding the present topic [30]. Overall, the evaluated journal metrics and the mapping analysis carried out with VOSviewer were classified as external quality indicators of the selected studies.

Thus, the findings of the present systematic review seem to represent a valid and relevant contribution to understanding and comprehending the most predominant factors of COVID-19 vaccine hesitancy at a global level, which remains a hot topic.

### 4.1. Global Predictors, Single Occurrences and Contradictory Predictors of Vaccine Hesitancy

#### 4.1.1. Global Predictors of Vaccine Hesitancy

Considering that the contradictory predictors of vaccine hesitancy (e.g., males vs. females) are likely to be influenced by the social, political and/or cultural characteristics of a particular country, the global predictors of vaccine hesitancy were discussed once these were excluded.

The top five most predominant predictors of vaccine hesitancy (after exclusion of the contradictory predictors) were: lower perceived risk of getting infected, a lower level of institutional trust, not being vaccinated against influenza, lower levels of perceived severity of COVID-19 infection and stronger beliefs that the vaccination would cause side effects or be unsafe. Importantly, three out of these five predictors were also identified as relevant factors in the two previous related/similar systematic reviews and meta-analyses by Wang et al. (2021) (influenza vaccination history and trust in the government) and Roy et al. (2022) (safety of COVID-19 vaccines) [19,20], which also supports the accuracy of the present systematic review.

However, other relevant factors have been identified in the present systematic review (i.e., lower perceived risk of getting infected and lower levels of perceived severity of COVID-19 infection), which may raise the awareness of other relevant explanatory variables/predictors of vaccine hesitancy in future research. For instance, belonging to a certain political party and having lower levels of perceived effectiveness/efficacy of a COVID-19 vaccine were also relevant predictors of vaccine hesitancy (after excluding the contradictory predictors). These findings are of the utmost relevance, since vaccination against influenza is not frequent in developing countries. Additionally, some contradictory predictors of vaccine hesitancy, such as belonging to a minority group or having a low income, may be more relevant for developing countries than for the developed ones. Overall, the number of studies carried out in developing countries was very limited. Thus, the top predictors are more likely to be explanatory variables of vaccine hesitancy in developing countries.

The subjects’ misperceptions, distress and scepticism can lead to COVID-19 vaccine hesitancy. Pervasive misinformation on COVID-19 (e.g., fake news or the spread of imprecise or false information about COVID-19 on social media, such as Facebook or other networks) is likely to explain some of the most relevant predictors of COVID-19 vaccine hesitancy identified here [95,96,97,98].

Additionally, the top five predictors identified here are covered in previous explanatory models of vaccine hesitancy (e.g., the 3C model and/or the 2017 increasing vaccination model) [14,16]. In general, these top five predictors are mainly related to the subjects’ opinions/perceptions, except for not being previously vaccinated against influenza (or being less willing to get a flu vaccine). Thus, health professionals or social workers should understand subjects’ opinions/perceptions about COVID-19 risks, severity and transmission, as well as the safety issues of COVID-19 vaccines, to clarify and explain potential misinterpretations. The intervention and/or consultation of health professionals was previously identified to aid in avoiding subjects’ vaccine hesitancy [56,99].

Other frequent predictors of vaccine hesitancy were being Republican or Conservative; having lower levels of perceived effectiveness/efficacy of COVID-19 vaccines; being single or non-married; having children at home; accepting vaccine conspiracies; having a lower level of worry about health risks; not shielding; being less likely to wear a mask; or having no family or friends with a previous COVID-19 infection (predictors reported in three or more studies). The preference for a certain political party was predominantly reported in the USA [59,60,61,62,64,65], although a study from South Korea also identified this predictor [86]. Thus, political parties/leaders should clarify the importance of vaccination to avoid the refusal of vaccines by their affiliates or sympathisers. Single or non-married people may be more worried about their health state since they live alone. People with children at home may believe that they will get COVID-19 through their children and therefore do not need to be vaccinated. People who accept vaccine conspiracies are less likely to trust in vaccines. A lower level of worry about health risks, not shielding and being less likely to wear a mask seem to indirectly support a lower perception of the potential risks of COVID-19, while the lack of COVID-19 infections in family or friends also seems to minimise the perceptions of the potential risks of this virus. However, these hypotheses should be demonstrated in future social studies by region/country.

#### 4.1.2. Single Occurrences

Single occurrences of predictors of vaccine hesitancy (i.e., predictors only reported/identified in one study) were very diverse/heterogeneous. These predictors should be evaluated in future research. It was not possible to check whether these variables have been tested in the construction of the administered questionnaires, since methodologies around the design, development or validation of questionnaires/surveys were incompletely described in the 37 selected studies. Thus, research methodologies relating to the development and validation of study questionnaires/surveys (i.e., collection tools) should be reported in more detail in future studies.

#### 4.1.3. Contradictory Predictors of Vaccine Hesitancy

Some of the contradictory predictors (i.e., contrary/opposite variables/predictors, which explain vaccine hesitancy in at least two countries/regions, such as females vs. males, or minorities vs. major ethnic groups) can be classified as potentially unexpected, as follows:

- Older people are more vaccine-hesitant than younger people, since the former are expected to be more susceptible to COVID-19 infection;
- Subjects with a higher level of education are more vaccine-hesitant than less educated subjects, since better-educated people are expected to be more prepared to understand the relevance of immunisation;
- Employed subjects are more vaccine-hesitant than unemployed subjects, since employed people are theoretically more likely to have more public contact;
- Citizens living in cities are more vaccine-hesitant than those living in rural areas or in smaller settlements (with a lower population density), since personal contact is less likely in rural areas.

These contradictory predictors of vaccine hesitancy are likely to be explained by political and/or cultural differences between countries/regions, although more studies are required to confirm this. The identification of contradictory predictors confirms the need to check the most prevalent explanatory variables of vaccine hesitancy per country/region, such as an eventual universal tool to determine subjects’ vaccine hesitancy based on the top five predictors, as the constitution of universal predictors of vaccine hesitancy is necessarily related to some limitations.

#### 4.1.4. Most Frequent Predictors of Vaccine Hesitancy per Country or Region

The most frequent predictors of vaccine hesitancy per country or region were variable (Table 6). For instance, the impact of belonging to a certain political party (i.e., parties other than the Democratic Party) on subjects’ vaccine hesitancy was predominately identified as an explanatory variable in the USA [59,60,61,62,64,65]. Thus, political leaders, affiliates and/or collaborators from parties other than the Democratic Party should ideally explain the relevance of immunisation to mitigate and control the COVID-19 pandemic in the USA. In opposition to the political preference of subjects, being a female was the most frequent predictor of vaccine hesitancy in almost all other reported regions/countries [60,61,62,64,67,69,74,75,76,77,80,81,82,83,88,89], which reinforces the importance of understanding females’ motivations about COVID-19 vaccination. Women are likely to benefit from health professional counselling and training on COVID-19 immunisation.

Younger people are expected to be more vaccine-hesitant than older individuals, since COVID-19 is less likely to be severe in the first group, although this variable was only identified as a frequent predictor of vaccine hesitancy in some countries/regions (e.g., the USA, some Asiatic countries and South Africa) [58,62,63,65,69,71,73,79,80,81,86,88,92]. Thus, younger people should be encouraged to get the COVID-19 vaccine to facilitate achieving herd immunity, mitigating the pandemic, controlling mortality, lethality and/or morbidity due to COVID-19. Contrary to expectation, older age was identified as one of the most frequent predictors of vaccine hesitancy in two studies from Latin America and/or the Caribbean, which is a concrete example of social and cultural variations between countries [89,90]. People from older groups can be less health-literate or educated in some countries (e.g., Mexico and Brazil), which may explain their higher levels of vaccine hesitancy in these nations.

Minorities and lower income were reported as the most explanatory variables of vaccine hesitancy for both the USA and South Africa [59,60,61,62,63,64,92], while a lower level of education was identified as one of the most frequent predictors of vaccine hesitancy in Latin America and South Africa [89,90,92]. These variables seem to be correlated: people from minority groups usually earn lower salaries, are less favoured and less educated than the general population. People from minority groups can also have language and/or health literacy limitations, which can limit their understanding of COVID-19 vaccination. These groups could also benefit from tailored vaccination campaigns and/or more pro-active healthcare interventions (e.g., physicians, nurses, pharmacists, social workers or others) [99].

Subjects’ perceptions about COVID-19 were only reported as the most predominant predictors of vaccine hesitancy in some countries (e.g., a perceived low risk of COVID-19 infection was identified in China and South Africa, and a lower perception of the severity of having COVID-19 was identified in Asiatic countries) [67,69,76,80,83,84,92]. Perceptions about COVID-19 vaccines were only reported as the most predominant predictor in three countries (perceived safety risks of COVID-19 vaccines in China and the UK, and perceived low efficacy of COVID-19 vaccines in China and South Africa) [66,67,68,92]. In this sense, subjects’ perceptions about COVID-19 vaccines and/or disease seem to be more relevant in some countries than in others as explanatory factors of subjects’ vaccine hesitancy. Health professionals should evaluate patients’ perceptions of COVID-19 and COVID-19 vaccination during consultations to help in the immunisation decision-making process [56].

The adequacy, perception and/or awareness of COVID-19 information (e.g., COVID-19 vaccination) were identified as the most frequent predictors of vaccine hesitancy in the UK and South Africa [71,72,92], which reinforces the need to develop clear and comprehensible written and oral health materials on COVID-19-related topics. This type of health material should be evaluated through usability tests, with the involvement of citizens from different sociodemographic backgrounds.

A lack of trust of public authorities/government or health systems (e.g., healthcare professionals) was reported as the most predominant predictor of vaccine hesitancy in Asiatic countries and in some European Union member states [76,79,83,84,87,88]. Thus, studies to understand the reasons why citizens do not trust in health institutions or healthcare professionals are highly recommended. The most advanced health systems in the world are among the member states of the European Union, in contrast to some Asiatic countries, although citizens motivations were not evaluated.

Not being vaccinated against influenza (UK) [71,72], older people (Latin America) [89,90], people with children at home (some European Union member states) [57,88], not having a partner (South Africa) [92] and not living in cities or living in city suburbs/smaller settlements (some European Union member states) [57,88] were examples of individual variables identified as the most relevant predictors of vaccine hesitancy in just one country/region, which strengthens the relevance of investigating potential social discrepancies within countries/regions. These findings support the development and implementation of studies on the present topic for individual countries/regions aiming to define tailored political health measures, since socio-demographic predictors are likely to vary with time.

According to the findings of the systematic review of Roy et al. (2022) [20], the most common predictors of vaccine hesitancy per country/region were: safety, efficacy, side effects, effectiveness and conspiracy beliefs (Asian countries); side effects, trust and social influence (Europe); and information sufficiency, political roles and vaccine mandates (United States). Globally, only two factors were common between the systematic review of Roy et al. (2022) [20] and the present systematic review: one in member states of the European Union (trust) and one in the USA (political roles), which confirms the likely variation in explanatory factors with time and/or an eventual lack of precision or sensitivity in the implemented studies in some countries/regions (e.g., potential limitations on the development and/or validation of study questionnaires/surveys about vaccine hesitancy/acceptance).

### 4.2. Study Strengths and Contribution to the State-of-the-Art on Vaccine Hesitancy

The selection criteria of the present systematic review were based on studies that use multivariate logistic regression models, different regions/countries and large samples (at least 868,742 subjects participated in the selected studies). Thus, the predictors of vaccine hesitancy were likely to be precisely and accurately determined in the present work. The same or similar selection criteria were not applied in previous similar or related systematic reviews. Considering that the predictors of vaccine hesitancy are expected to longitudinally vary within the same country or between different countries, health authorities are required to regularly evaluate vaccine hesitancy, especially in countries with high rates of vaccine hesitancy.

Moreover, the contradictory predictors are exhaustively explained, and a detailed list of predictors of vaccine hesitancy is presented. These findings will support the development of new international studies on vaccine hesitancy and evaluation tools. In opposition to the published studies, both the most and least relevant predictors of vaccine hesitancy are described and explained. The methodologies and questionnaires of the selected studies are heterogeneously defined. Therefore, the present systematic review will contribute to strengthening and improving the methods of future research on vaccine hesitancy.

Finally, at least two out of the five most relevant predictors (i.e., the participants’ lower perceived risk of getting infected and the lower perceived severity of COVID-19 infection) were not previously identified as the most relevant, reinforcing the need to evaluate subjects’ perceptions of COVID-19 risks. The fact that three out of the five most relevant predictors of vaccine hesitancy were common between the present systematic review and the two previous similar/related systematic reviews (i.e., a lower level of institutional trust, not being vaccinated against influenza and stronger beliefs that the vaccination would cause side effects or be unsafe) seems to be an external validity indicator of the quality of the present systematic review.

Overall, the findings of the present systematic review clearly contribute to enhancing the state of the art on vaccine hesitancy.

### 4.3. Risk of Biases and Limitations of the Present Systematic Review

There is ultimately a risk of selection bias, since more screened databases (e.g., Scopus and Embase) and keywords could have been used, and a risk of interpretation bias, since the impact of vaccination campaigns on subjects’ opinions/perceptions of vaccine acceptance/hesitancy have not been specifically evaluated (e.g., the findings from studies that started after and/or before the beginning of COVID-19 vaccination were analysed together).

There is risk of data collection bias, as data were only collected, checked and rechecked by just one author, which is not in line with the 2020 PRISMA recommendations [22]. However, all data were at least double-checked, and an external validation was carried out, given that three out of five of the most relevant predictors of vaccine hesitancy were equal/common with those identified in two previous similar/related systematic reviews [19,20]. This achievement seems to confirm the study accuracy and validity. Additionally, other methodological procedures were adopted to avoid data collection imprecisions, such as the adopted data collection methodologies. For instance, study objectives were purposively not rephrased to avoid any inaccuracy (Appendix A), although the remaining information was rephrased to avoid plagiarism.

Moreover, automation tools, such as SRDR, Distiller SR or Dedoose, were not used to automatically collect and process information [100,101,102]. To avoid potential bias in the analyses, more authors or automatic tools could have been involved in the design and execution of the present systematic review (e.g., to reconcile divergent points of view and to achieve consensus).

Subgroups analysis and sensitive analysis are not the same, although both contribute to study quality and validity. Besides the evaluation of the most frequent predictors of vaccine hesitancy per country or region and the evaluation of study findings after excluding contradictory predictors of vaccine hesitancy (subgroups analysis), a specific sensitivity analysis was not carried out in the present systematic review. Considering that the global rating of the NHLBI quality assessment tool was 82.1% (fair), an eventual exclusion of specific studies/findings based on the risk of bias did not seem to be relevant/applicable.

Additionally, the definition of quantitative thresholds or cut-off values is not possible, since qualitative predictors of vaccine hesitancy were calculated based on a multivariate regression model in the present systematic review. For instance, specific thresholds can be defined for certain biological parameters (e.g., glycaemia) in sensitive analysis of clinical studies.

A possible sensitivity analysis could have been based on the exclusion of studies applying different data collection tools (e.g., surveys using different questions or scales) or utilising different types of administration (e.g., self-administered, presential, online questionnaires, etc.). However, this evaluation was not performed, because the questionnaires from the selected studies were very heterogeneously designed (e.g., different questions, scales/scoring or administration types). Moreover, questionnaires were insufficiently described in some of the selected studies.

A protocol was not previously registered, such as in the Open Science Framework (OSF) repository [103]. However, the initial protocol has not been changed, all methodological details are described here, and the present systematic review does not involve clinical trials or epidemiological studies of medicines (i.e., there are no ethical issues).

### 4.4. Limitations and Risk of Biases of the Selected Studies

The selected studies were heterogeneously designed. For instance, the evaluated variables/collected data were variable between different studies (e.g., subjects’ perceptions of COVID-19 or opinion of COVID-19 vaccines were only evaluated in some studies).

The type of collected data and questionnaires were variable between the selected studies, including different collected topics and the order of questions. The different order of questions may have introduced question order bias [104,105]. Thus, some studies may not be precise or sensitive. Studies on the present topic were only developed in some countries/regions (i.e., USA, China, UK, Australia, some Asiatic countries, the European Union, three countries from Latin America and the Caribbean and South Africa). The potential disadvantages of COVID-19 vaccination were not discussed in detail (e.g., the impact of adverse drug reactions on patients’ health, limited efficacy and/or number of required shots). The questionnaire/survey construction and validation should be clearly described in the methods, since questionnaire-based methodologies were, in general, insufficiently reported in the selected studies.

Additionally, studies based on web-based surveys may have introduced bias into the results, since the profile and sociodemographic characteristics of the non-participants/respondents may have been imprecisely collected (e.g., data collection in social networks). Studies applying triangulation methodologies were limited (e.g., administration of additional tools, such as health literacy, numeracy or cognitive evaluation tools, tools to evaluate subjects’ quality of life, social life, satisfaction and/or wellbeing (before and after the beginning of the pandemic), evaluation of knowledge about COVID-19 or vaccines, perception evaluations or simulations of decisions based on imaginary scenarios). Some variables were only quantified in some studies, such as the number/proportion of vaccinated individuals, perception of the safety and efficacy of COVID-19 vaccines and opinions about the severity of COVID-19, among other factors/variables. The sociodemographic variables were not distributed in a balanced way in all selected studies (e.g., the proportion of females/males or education level), although multivariate models normalise the contribution of individual variables in study findings. Longitudinal studies were lacking in most countries.

The selected studies were carried out in three timeframes (before and after the beginning of COVID-19 vaccination and/or involving both timeframes), which may have influenced the study findings. Moreover, when comparing the studies carried out before (first group) and after (second group) the beginning of COVID-19 vaccination, the willingness to get a COVID-19 vaccination was above 50% in all studies in the second group. Importantly, few studies from the first group reported a willingness to get a COVID-19 vaccine below 50% [80,84] (Appendix A). In this sense, COVID-19 hesitancy may have tended to decrease following the start of COVID-19 vaccination. However, data are not conclusive, since studies were generally only carried out in just one period, and there was a discrepancy between the type of questionnaires/collection tools used in the selected studies. The present findings reinforce the need to design and implement more prospective and/or retrospective studies on COVID-19 vaccine hesitancy.

According to the findings of the NHLBI tool, a selection bias may have occurred in some of the selected studies, since the participation rate and the profile of non-respondents (e.g., sociodemographic characteristics) were not reported in most studies. Information about study representativeness, power description or variance was only provided in around half of the selected studies, although at least 500 participants were enrolled. On the other hand, authors of the selected studies may have calculated the sample size based on power description or variance but may have opted not to publish this information. As sample size calculations were not reported in many studies, it is not possible to exclude the occurrence of study bias because of imprecise sample calculations. In general, questionnaires were only administered once. Thus, participants’ perceptions on the willingness to get a COVID-19 vaccines were not checked/followed during a certain timeframe in almost all selected studies.

### 4.5. Future Research and Purposed Intervention Measures

The present research should involve more countries/regions. More studies and international collaborations are recommended to understand the contradictory predictors of vaccine hesitancy (e.g., females vs. males or minorities vs. major ethnic groups). An international tool to evaluate vaccine hesitancy could be developed and validated, based on the most frequent predictors of vaccine hesitancy reported in the present systematic review, for instance. High-quality health-related information about COVID-19 vaccines and immunisation should be developed and evaluated through usability tests in groups of participants from different sociodemographic backgrounds. Social studies are recommended to understand why citizens do not trust public authorities/government or health systems, or why they believe in conspiracy theories. Health authorities should develop detailed guidelines about the development and validation of data collection tools to characterise subjects’ vaccine hesitancy (e.g., surveys or questionnaires), preferably per country/region. Sensitivity analyses are recommended in future research.

Health communication strategies seem to be a factor in understanding and reducing vaccine hesitancy. For instance, mass media, apps, video games and/or social networks can divulge high-quality information on the risk of being infected with COVID-19, the severity of COVID-19 infection, or the safety of COVID-19 vaccines, since these topics are associated with the most relevant predictors of vaccine hesitancy.

Additionally, health professionals (physicians, pharmacists, or nurses) can try to understand the subjects’ perceived risks of becoming infected with COVID-19, institutional trust, influenza vaccination state (vaccinated against influenza or not), perceived severity of COVID-19 infection or opinions about the safety of COVID-19 vaccines, in order to understand the motives behind vaccine hesitancy. If applicable, health professionals should clarify subjects’ doubts and inaccurate perceptions/opinions. In general, health professionals’ counselling and intervention can effectively contribute to mitigating subjects’ vaccine hesitancy [99]. National information campaigns are also recommended, covering all community pharmacies and/or health centres for instance, since health professionals are prepared to clarify citizens’ doubts. Ideally, health professionals should receive training on communication strategies to effectively convey all health messages. These health messages should be adapted/tailored according to the level of the subjects’ health literacy.

## 5. Conclusions

Globally, 37 questionnaire-based studies on the predictors of vaccine hesitancy were selected, which were estimated through multivariate regression models. The selected studies covered a significant number of countries/regions and respondents. These studies complied with satisfactory internal quality measures (e.g., the outputs of the NHLBI assessment tool) and external quality standards (e.g., bibliographic metrics or mapping analysis), which seems to confirm the relevance/quality of the present systematic review. However, the implementation of similar studies is still lacking in many countries at a global level.

The five most predominant predictors of vaccine hesitancy (after exclusion of contradictory predictors) were: a lower perceived risk of getting infected, lower levels of institutional trust, not being vaccinated against influenza, a lower perceived severity of COVID-19 infection and stronger beliefs that the vaccination would cause side effects or be unsafe. These findings were accurately determined through the application of multivariate regression models, which support their possible application in international research (e.g., the validation of a future tool to quickly detect COVID-19-vaccine-hesitant subjects). These top five predictors can also be used to design tailored health policies and/or public health interventions.

Diverse contradictory predictors have been identified (i.e., contrary variables/predictors, which explain vaccine hesitancy in at least two countries/regions, such as females vs. males, or minorities vs. major ethnic groups). Additionally, the profile of the most frequent predictors of vaccine hesitancy also varied between analysed countries/regions. Globally, these findings confirm the likely existence of social cultural differences between countries/regions and the need to check the profile of these predictors per country/region.

However, while two previously identified similar/related systematic reviews did not apply the same inclusion/exclusion criteria (e.g., the obligatory calculation of multivariate logistic regression modes to estimate the predictors of vaccine hesitancy/acceptance), they reported some common frequent predictors of vaccine hesitancy [19,20]. These findings seem to confirm the validity and relevance of the present systematic review and, indirectly, the accuracy of the implemented research. A lower perceived risk of getting infected and a lower perceived severity of COVID-19 infection were among the most frequent explanatory predictors of vaccine hesitancy, especially in the present systematic review.

The selected studies covered a broad number of countries/regions (i.e., USA, China, UK, Australia, Asiatic countries, member states of the European Union, Latin America and the Caribbean and South Africa) and enrolled a significant number of participants, which strengthens the relevance of the study conclusions.

## Figures and Tables

**Table 1:** PICOS: inclusion and exclusion criteria.

PICOS Criteria	Inclusion Criteria	Exclusion Criteria
P: participants	The general population (at least one country).	Studies specifically on specific subgroups (e.g., healthcare professionals or people with a certain disease, such as diabetes or asthma) were excluded.
I: intervention	Questionnaire-based studies, i.e., administration of a questionnaire/survey to collect participants’ opinion/perception about COVID-19 vaccine hesitancy/acceptance.	Studies about other topics and/or that were not questionnaire-based.
C: comparison	When applicable (e.g., studies carried out in more than one country).	
O: outcomes	To estimate the predictors of vaccine acceptance or vaccine hesitancy through a multivariate regression model.	Studies not estimating the predictors of vaccine acceptance or vaccine hesitancy through a multivariate regression model.
S: study design	Descriptive national studies, or descriptive studies enrolling at least 500 participants from the general population and carried out at a national level.	Studies enrolling less than 500 participants from the general population.

**Table 2:** Previous similar or related systematic reviews on predictors of vaccine hesitancy/acceptance.

Previous Identified Similar/Related Reviews	Covered Timeframe and Inclusion/Exclusion Criteria; Keywords and Number of Selected Studies	Main Findings and Conclusions
(Wang et al., 2021) [19] A systematic review and meta-analysis	Beginning of pandemic up to 4 November 2020 Inclusion criteria: The types of included studies were not limited. Studies that did not involve COVID-19 vaccine acceptance or did not provide specific survey numbers for pooling were excluded. Keywords: “COVID-19” OR “SARS-CoV-2” OR “novel coronavirus” OR “coronavirus disease 2019” AND “vaccin *” OR “immunization”, with (*) being used to automatically screen similar/derived words. Number of selected studies: 38	The stronger predictors of COVID-19 vaccination willingness were gender, educational level, influenza vaccination history and trust in the government.
(Roy et al., 2022) [20] Systematic review	Beginning of pandemic up to July 2021 Inclusion criteria: (1) peer-reviewed published articles from electronic databases including PubMed (MEDLINE), Elsevier, Embase, Science Direct, Scopus and other reputable resources; (2) survey studies involving all types of sample populations; (3) the scope and principal aim of the study was to identify the potential factors influencing COVID-19 vaccine acceptance and hesitancy; (4) publication studies in the English language. Keywords: “COVID-19 vaccine hesitancy” OR “COVID-19 vaccine hesitancy and associated factors” OR “COVID-19 vaccine confidence” OR “COVID-19 vaccine AND acceptance intention”. Number of selected studies: 47	The most common predictors of vaccine acceptance were as follows: safety, efficacy, side effects, effectiveness and conspiracy beliefs (Asian countries); side effects, trust in vaccine and social influence (Europe) and information sufficiency, political roles and vaccine mandates (United States).

* is a bolean operator used in some browsers to automatically screen for related/derived words.

**Table 3:** Dates of the first authorised or administered COVID-19 vaccine per each studied country.

Country	Date of the First Authorised or Administered COVID-19 Vaccine	COVID-19 Vaccine
Australia [33]	25 January 2021	Pfizer-BioNTech COVID-19 Vaccine (Comirnaty)
Brazil [34]	17 January 2021	CoronaVac, of the Butantan Institute, in partnership with Chinese pharmaceutical company Sinovac and AstraZeneca, of the Oswaldo Cruz Foundation (Fiocruz), in collaboration with the Astrazeneca/Oxford consortium
China [35]	30 December 2020	Sinopharm China Biotechnology Co., Ltd.
European Union (e.g., Italy; Croatia; France; Germany; Sweden or Spain) [9]	21 December 2020	Pfizer-BioNTech COVID-19 Vaccine (Comirnaty)
Hong Kong [36]	January 2021	Pfizer-BioNTech COVID-19 Vaccine (Comirnaty)
Indonesia [37]	11 January 2021	CoronaVac, from Sinovac Biotech China
Israel [38]	20 December 2020	Pfizer-BioNTech COVID-19 Vaccine
Japan [39]	14 February 2021	Pfizer-BioNTech COVID-19 Vaccine (Comirnaty)
Jordan [40,41]	13 January 2021	Pfizer-BioNTech and China’s Sinopharm coronavirus vaccines
Kingdom of Saudi Arabia [42]	17 December 2020	Pfizer-BioNTech COVID-19 Vaccine (Comirnaty)
South Korea [43]	10 February 2021	AstraZeneca COVID-19 vaccine
Lebanon [44]	24 March 2021	AstraZeneca vaccine
Mexico [45,46]	24 December 2020	Pfizer-BioNTech COVID-19 Vaccine (Comirnaty)
Norway [47]	21 December 2020	Pfizer-BioNTech COVID-19 Vaccine (Comirnaty)
Pakistan [48]	8 May 2021	Oxford-AstraZeneca COVID-19 vaccines
Qatar [49]	21 December 2020	Pfizer-BioNTech COVID-19 Vaccine (Comirnaty)
South Africa [50]	16 March 2021	Pfizer-BioNTech COVID-19 Vaccine (Comirnaty)
Trinidad and Tobago [51]	30 March 2021	AstraZeneca/Oxford vaccine, manufactured by SK Bioscience of South Korea
UK [52]	2 December 2020	Pfizer-BioNTech COVID-19 vaccine (Comirnaty)
United Arab Emirates [53]	9 December 2020	Sinopharm, Beijing Institute of Biological Products’ inactivated vaccine
USA [54]	11 December 2020	Pfizer-BioNTech COVID-19 vaccine (Comirnaty)

**Table 4:** Quality assessment of the 37 selected studies with the NHLBI tool [32].

Criteria	Score	Maximum Score	%
Was the research question or objective in this paper clearly stated?	37	37	100
Was the study population clearly specified and defined?	36	37	97.3
Was the participation rate of eligible persons at least 50%?	24 **	37	64.9 *
Were all the subjects selected or recruited from the same or similar populations (including the same time period)?	18.5	18.5	100
Were inclusion and exclusion criteria for being in the study prespecified and applied uniformly to all participants?	18.5	18.5	100
Was a sample size justification, power description or variance and effect estimate provided?	25	37	67.6 *
Were the exposure measures (independent variables) clearly defined, valid, reliable and implemented consistently across all study participants?	37	37	100
Was the exposure(s) assessed more than once over time? (i.e., Was the questionnaire administered at least two times in different moments?/exposure or evaluation of independent variable)	2	18.5	10.8 *
Was loss to follow-up after baseline 20% or less? (i.e., % of participants who did not reply to the second questionnaire)	1	18.5	5.4 *
Were the outcome measures (dependent variables) clearly defined, valid, reliable and implemented consistently across all study participants?	37	37	100
Were key potential confounding variables measured and adjusted statistically for their impact on the relationship between exposure(s) and outcome(s)?	37	37	100
Was loss to follow-up after baseline 20% or less?	37	37	100

* Poor scores (below 80%); ** the participation rate was not reported in 20 out of the 37 studies.

**Table 5:** Global predictors of vaccine hesitancy.

Predictors of Vaccine Hesitancy *	n **	% ***
Females	16	43.2
Younger/young and middle-aged	13	35.1
Lower perceived risk of getting infected/lower perceived risk of infection/lower fear to experiencing COVID-19 infection/perceiving infection as a low risk	12	32.4
Lower level of institutional trust of government, Ministry of Health and physicians or health system/lower trust in healthcare system or vaccine manufacturers/not valuing doctor’s recommendations/not trusting in medical sectors to manage COVID-19/not trusting in medical and scientific experts (e.g., WHO or national advisors)/not believing that the public authorities are handling the pandemic adequately/not trusting information provided by authorities	10	27.0
Not being vaccinated against influenza/were less willing to have a flu vaccine/not willing to get flu vaccines	9	24.3
Non-White or other minorities/non-Latinx Black/Black and Hispanic/Black, Asian and minority ethnic/other minorities, such as Arabs or non-Black African population group /migrant	8	21.6
Lower levels of perceived severity of COVID-19 infection/perceiving the severity of COVID-19 as a lower threat/not believing that COVID-19 can be debilitating and dangerous to health/lower levels of worry about the COVID-19 virus/perceiving the effect of the disease to have a lower effect on one’s personal health	8	21.6
Stronger beliefs that the vaccination would cause side effects or be unsafe/the risk of vaccines/higher levels of potential vaccine harm/stronger beliefs that the vaccine is unsafe/vaccine side effects	8	21.6
Lower level of education/less educated/persons with lower levels of education	7	18.9
Lower income/decreased family income	7	18.9
Republicans/Conservative/different party other than Democratic Party/not trusting in President Biden/living in a Republican-“leaning” state	7	18.9
Lower levels of perceived effectiveness of a COVID-19 vaccine/value of efficacy/assigning importance to the vaccine´s efficacy/lower perceived efficacy of vaccine/not believing in the vaccine’s ability to control the pandemic	6	16.2
Males	5	13.5
Non-married/single/without partners	5	13.5
Not residing in state capital/not living in cities or city suburb/living in a rural residential area/smaller settlements	5	13.5
Older	4	10.8
People with children at home/having children	4	10.8
Accepting vaccine conspiracies/they did perceive COVID-19 to be a hoax/viewing COVID-19 risks as exaggerated or believing that COVID-19 does not exist	4	10.8
Other major ethnic group (e.g., Emiratis or UAE nationals)/Arab ethnicity	3	8.1
Higher education/tertiary education (if spending more time using social media)/postgraduate	3	8.1
Unemployed/being self-employed, unemployed or unable to work due to a long-term illness or disability	3	8.1
Lower level of worry regarding health risks/complacency in health/not declaring to be concerned about one’s own health and the health of next of kin	3	8.1
Not shielding/less likely to wear masks	3	8.1
Not reporting previous exposure to COVID-19 among close persons/without COVID-19 infection in family or friends/infected by COVID-19 personally or within their family	3	8.1
White as major ethnic group	2	5.4
Employed	2	5.4
No chronic disease/no underlying medical conditions	2	5.4
Lower self-reported health outcomes	2	5.4
Not trusting in any source of information on COVID-19 vaccines/not trusting in the reliability of media sources regarding COVID-19	2	5.4
Not being a healthcare professional/not being a medical student	2	5.4
Insufficient perceived information to make an informed decision about COVID-19 vaccination/adequacy of information about the vaccine	2	5.4
Low frequency of attention to relevant COVID-19 information/lower awareness of COVID-19-related information	2	5.4
A higher endorsement of the notion that only people who are at risk of serious illness should be vaccinated for COVID-19/not believing that everyone should be vaccinated	2	5.4
Lower general vaccine knowledge/lower general vaccine knowledge index	2	5.4
People who do not have a positive view of the COVID-19 vaccine/lack of confidence in the COVID-19 vaccine	2	5.4

* Similar/equal predictors variables were aggregated, but the original designations were maintained to ensure study precision; ** number of occurrences in the 37 studies; *** 100% (n = 37).

**Table 6:** The most frequent predictors of vaccine hesitancy per country or region.

	n	%
USA (n = 7 Studies, 100%)		
Republicans/Conservative/different party other than Democratic Party/not trusting in President Biden/living in a Republican-“leaning” state	6	85.7
Females	4	57.1
Minorities	4	57.1
Younger	3	42.9
Lower income	3	42.9
**China (n = 4 studies, 100%)**		
Females	2	50
Perceiving low risk of infection	2	50
COVID-19 vaccines are unsafe	2	50
Lower efficacy of COVID-19 vaccines	2	50
**UK (n = 3 studies, 100%)**		
Not being vaccinated against influenza	2	66.7
COVID-19 vaccines are unsafe	2	66.7
Insufficient perceived information to make an informed decision about COVID-19 vaccination or adequacy of information about the vaccine	2	66.7
**Australia (n = 3 studies, 100%)**		
Females	2	66.7
**Asiatic countries (n = 11 studies, 100%)**		
Females	6	54.5
Younger	4	36.4
Lower level of institutional trust of public authorities/government or health system	4	36.4
Not believing that COVID-19 can be debilitating and dangerous to health/lower perceived risk of getting infected/lower perceived severity of having COVID-19	4	36.4
**European Union, Italy and Croatia (n = 3, 100%)**		
People with children at home	2	66.7%
Lower level of institutional trust of public authorities/government or health system	2	66.7%
Not living in cities or living in smaller settlements	2	66.7%
**Latin America and Caribbean (n = 3, 100%)**		
Older	2	66.7%
Persons with lower levels of education	2	66.7
**South Africa (n = 1, 100%)**		
Lower perceived risk of infection	1	100%
Lower perceived efficacy of vaccine	1	100%
Lower awareness of COVID-19-related information	1	100%
Lower income	1	100%
Minorites	1	100%
Younger	1	100%
Less educated	1	100%
Without partner	1	100%

n: number of studies.

* Only some of the collected sociodemographic variables are reported here due to space limitations; ** studies that not exclusively evaluate predictors of vaccine acceptance and/or hesitancy.

## Data Availability

The main findings are available in the present article.

## Appendix A. Main Findings of the X Selected Studies

**Table:**

Author, Year, Geographic Region, Database; Journal (Impact Factor JCR 2021 and Quartile SJR 2021)	Study Aim	Sample Size (Number of Participants that Completed the Study, i.e., Valid Respondents) and Main Characteristics *	Methods; Date of the Administration of the Questionnaire	Findings	Discussion and Conclusion
**Studies involving more than one country**					
(Kerr et al., 2021) [56] Twelve countries: Australia, China, France, Germany, Italy, Japan, Korea, Mexico, Spain, Sweden, UK, USA Bon ***BMJ Open*** **(IF JCR = 3.006; Q1)**	*To describe demographical, social and psychological correlates of willingness to receive a COVID-19 vaccine.*	25,334 respondents Age (mean = 45.06); female (mean = 0.51); Conservative (mean = 3.74); general trust: experts (mean = 3.97).	Online surveys Multivariate logistic regression March and October 2020 (before the beginning of the COVID-19 vaccination roll-out).	Reported willingness to receive a vaccine varied widely across samples (63% to 88%). Explanatory variables of vaccine hesitancy: male, not trusting in medical and scientific experts (e.g., WHO or national advisors), lower levels of worry about the COVID-19 virus, age, not being prosocial and low perceived infection risk. In particular, the strongest predictors of vaccine acceptance among the sampled countries were being a female, trust in medical and scientific experts and worry about the COVID-19 virus.	Information provided by medical and scientific experts (credible sources) seems to be determinant of vaccine acceptance.
(Mascherini and Nivakoski, 2022) [57] European Union Bon ***Vaccine*** **(IF JCR = 4.169; Q1)**	*To examine COVID-19 vaccine hesitancy in the European Union, focusing on the role of social media use*	29,755 respondents Female (63.8%); ≥60 years (33.7%); living in a city or city suburb (39.5%); employed (49.2%); tertiary education (66.3%); lives with spouse (61.8%); children in household (34.8%); bad health (8.3%); chronic health problem (46%); close person had COVID-19 (38%).	Cross-national survey covering all 27 EU member states Multivariate regression models February and March 2021 (after the beginning of COVID-19 vaccination roll-out).	A total of 71.3% respondents were very likely or rather likely to get the COVID-19 vaccine. Explanatory variables of vaccine hesitancy: men, not living in cities or city suburbs, being self-employed, unemployed or unable to work due to a long-term illness or disability, students, singles (not living with a spouse or partner), living with children in the household, tertiary education (if spending more time using social media), not reporting previous exposure to COVID-19 among close persons, viewing COVID-19 risks as exaggerated—or believing that COVID-19 does not exist (more likely to be male and in very good health), people who use social media for 3 or more hours daily, people who report social media as their main source of news.	Clear, precise and transparent messages about vaccines need to be delivered by policymakers and scientists, namely in social media. For instance, men, people in good health and those using social media as their main source of news are more likely to consider COVID-19 risks to be exaggerated (or that COVID-19 does not exist).
(Price et al., 2022) [58] Norway, the UK, the USA and Australia Bon ***Social Sciences*** **(no IF JCR; Q2; available in Web of science and Scopus)**	*To examine (i) the willingness in the general population to get the COVID-19 vaccine nine months after the pandemic outbreak and (ii) the willingness to get the vaccine in relation to sociodemographic variables, whether one has experienced COVID-19 infection, concerns about health and family and trust in the information provided by authorities about the pandemic.*	3474 respondents Female (66.8%); 18–29 years (69.8%); city (70.4%); master’s/doctoral degree (72.5%); full-time or part-time employed (66.3%); infected (55.1%); trust in public information provided by authorities (79.4%).	Cross-sectional survey Logistic regression analysis 24 October and 29 November 2020 (before the beginning of COVID-19 vaccination roll-out).	A total of 65% respondents reported being likely or very likely to get the COVID-19 vaccine (USA 63%, UK 66.6%, Norway 69.5%, and Australia 71.1%). Explanatory variables of vaccine hesitancy: male, younger people, not living in a city, unemployed, low education level (high school or lower), infected by COVID-19 personally or infection within their family, not declaring to be concerned about their own health and the health of next of kin and not trusting information provided by authorities. The trust in the information provided by authorities about COVID-19 is an especially relevant predictor, since a significantly higher vaccine acceptance was shown across all countries.	Information provided by authorities should be used to increase the proportion of citizens willing to get the COVID-19 vaccine. Study findings should be used in future vaccination campaigns and public health measures.
**USA**					
(Reiter et al., 2020) [59] USA *PubMed* ***Vaccine*** **(IF JCR = 4.169; Q1)**	*To examine the acceptability of a COVID-19 vaccine among* *a national sample of adults in the US.*	2006 adults 43% Female; White, non-Latinx (67%); married/civil union or living with partner (51%); high school degree (29%); Conservative (33%); no health insurance (12%); underlying medical condition, yes (36%).	Online survey Multivariable relative risk regression May 2020 (Before the beginning of COVID-19 vaccination period).	69% of respondents were willing to get a COVID-19 vaccine. Explanatory variables of vaccine hesitancy: if respondents though their healthcare provider would not recommend vaccination); Conservative (political party), lower levels of perceived likelihood of getting infected with COVID-19 in the future; lower levels of perceived severity of COVID-19 infection; lower levels of perceived effectiveness of a COVID-19 vaccine; non-Latinx Black; and higher levels of potential vaccine harms.	These findings can Help in guiding and planning the development of future public health efforts to increase the acceptability of COVID-19 vaccines.
(Khubchandani et al., 2021) [60] USA *PubMed* ***Journal of Community Health*** **(IF JCR = 4.371; Q1)**	*To conduct a comprehensive* *and systematic national assessment of COVID-19 vaccine hesitancy in a community-based sample of the American* *adult population.*	1878 adults Females (52%), less than 40 years (63%); White (74%), non-Hispanic (81%), married (56%), employed full time (68%) and has a bachelor’s degree or higher (77%).	A multi-item validated questionnaire. Logistic regression model analysis. June 2020 (before the beginning of COVID-19 vaccination period).	Likelihood of getting a COVID-19 immunisation: very likely (52%), somewhat likely (27%), not likely (15%), definitely not (7%). Explanatory variables of vaccine hesitancy: females, lower levels of education, employed people, lower income, people with children at home, Republicans, and people who perceived the likelihood of getting infected in the next 1 year as “definitely not”.	Special attention to the groups identified in this study as vaccine-hesitant. It is recommended that evidence-based communication interventions, such as mass media strategies and/or policy measures, are defined.
(Ruiz and Bell, 2021) [61] USA *PubMed* ***Vaccine*** **(IF JCR = 4.169; Q1)**	*To assess* *the impact of people’s main media source of COVID-19 information* *on vaccine hesitancy.*	804 English-speaking adults. Female (53.6); White race (65.3%); less than 45 years (51.6%); married/living as married (56.6%); postgraduate degree (23.3%); Democrat (31.1%); preferred media for coronavirus news (1st CNN (17.5%); 2nd Fox News (16.3%) and 3rd ABC (14.1%).	Internet survey Multiple linear regression. 15–16 June 2020 (before the beginning of COVID-19 vaccination period).	62.2% of respondents were extremely or somewhat likely to get COVID-19 vaccine. Explanatory variables of vaccine hesitancy: lower general vaccine knowledge; accepting vaccine conspiracies; perceiving the severity of COVID-19 as a lower threat; no influenza vaccine uptake in the current flu season; less than 5 pre-existing health conditions that make one susceptible to COVID-19; female; an income of less than USD 120,000; party than Democrat; relying upon social media for virus information; intent to get vaccinated was lower for Fox News (57.3%) than CNN/MSNBC viewers (76.4%)) (social media virus information).	The present study contributes to guide public health experts, regarding the development of the strategies for encouraging uptake of COVID-19 vaccines.
(Latkin et al., 2021) [62] USA *PubMed* ***PLoS One*** **(IF JCR = 3.752; Q1)**	*To examine the prevalence of COVID-19 vaccine hesitancy and factors* *associated with vaccine intentions.*	1056 respondentsFemale (70.1%); more than 59 years (43.3%); White (69.%); some college and above (80.5%); employed (56.7%); USD 50,000 or more (57.1%); not at all worried about COVID-19 infecting family or self (7.2%); Conservative (28%).	A national panel survey (telephone and web) Multinominal logistic regression 14 and 18 May 2020 (before the beginning of COVID-19 vaccination period).	53.6% participants reported intending to be vaccinated. Explanatory variables of vaccine hesitancy: black and Hispanic; females; younger respondents; and political party (Conservative). More vaccine-hesitant respondents were significantly less likely to report that they engaged in the COVID-19 prevention behaviours (e.g., wearing masks).	Campaigns should address study findings, i.e., assess in greater detail the vaccine concerns of Blacks, Hispanics and women to tailor programs.
(Nguyen et al., 2021) [63] USA Bon ***Vaccine*** **(IF JCR = 4.169; Q1)**	*To understand disparities in vaccine intentions and reasons for vaccine hesitancy.*	459,235 respondents Female (51.6%); ≥65 years (21.7%); White (62.5%); some college or college graduate (47.7%); <USD 35,000 (19.3%); insured (91.7%).	Census Bureau’s Household Pulse Survey Logistic regression Models 6 January –29 March 2021 (After the beginning of COVID-19 vaccination period).	On average, 62% of respondents reported being willing to receive at least one COVID-19 vaccine. Explanatory variables of vaccine hesitancy: non-Hispanic Black adults with lower income (<USD 35,000) and younger age (18–49 years).	Interventions and recommendations to improve vaccination coverage and confidence should be designed to target some populational groups, such as younger and lower income racial/ethnic minority groups.
(Omaduvie et al., 2021) [64] USA Bon ***Population Medicine*** **(no impact factor JCR; indexed in Scopus)**	*To examine whether the wide variations in the burden of COVID-19 incidence and mortality rates across states are associated with vaccine hesitancy.***	68348 respondents Female (51.6%); age (mean = 47.2 years); non-Hispanic White (62.6%); married (55.1%); college degree (26.7%); annual household income (USD) less than 25,000 (14.4%).	Household Pulse Survey Multivariable Poisson regression model. 6–18 January 2021 (After the beginning of COVID-19 vaccination period).	23.5% reported vaccine hesitancy. Explanatory variables of vaccine hesitancy: being Black, having already tested positive for COVID-19, female, and living in a Republican-“leaning” State.	Knowledge of state-specific information can be useful to define specific intervention programs.
(Hao et al., 2022) [65] USA Bon ***Vaccine*** **(IF JCR = 4.169; Q1)**	*To understand public behaviour regarding COVID-19 vaccination.*	Approximately 6000 respondents Females (59%); age (mean =53); White (78%); married (56%); employed (51%); Democrat (30%); vaccinated for the coronavirus (72%).	National survey Multilevel logistic regression 9 June and 21 July 2021 (after the beginning of COVID-19 vaccination period ).	On average, around 72% of respondents declared to have gotten vaccinated for the coronavirus. Explanatory variables of vaccine hesitancy: not trusting in President Biden, lower proportion of their family or close friends have already received it, people who do not have a positive view of the COVID-19 vaccine, lower age, White, not married.	Study finding contribute to understand the profile of citizens who do not accept the vaccine, which can be helpful to design new programs/interventions.
**China**					
(Dong et al., 2020) [66] China *PubMed* ***Health expectations*** **(IF JCR = 3.318; Q1)**	*To examine how factors* *related to vaccine characteristics, their social normative influence and the convenience* *of vaccination can affect the public’s preference for the uptake of the COVID-19* *vaccine in China.*	1236 respondents Female (50.89%); age (mean = 30.27; SD = 7.66); unmarried (38.83%); unemployed (0.97%); education: tertiary and above (85.76%).	Survey: an online discrete choice experiment A mixed logit regression model. June and July 2020 (before the beginning of the COVID-19 vaccination period).	Explanatory variables of vaccine hesitancy: lower efficacy (less than 70% or 90%); shorter duration of action (less than 12 or 18 months); more adverse events, number of shots (more than one injection); production place (non-imported vaccines); and price (higher price).	Identified preferences should be used to develop COVID-19 vaccination programs in China.
(Wang et al., 2020) [67] China *PubMed* ***Vaccines*** **(IF JCR = 4.961; Q1)**	*To evaluate* *the acceptance of COVID-19 vaccination in China and give suggestions for vaccination strategies* *and immunisation programs accordingly.*	2058 adults Female (54.2%); 41 or more years (32.1%); more than high school (61.8%); married (67.3%).	Anonymous cross-sectional Survey (online) Multivariate logistic regression March 2020 (before the beginning of the COVID-19 vaccination period).	91.3% respondents reported that they would accept COVID-19 vaccination after the vaccine becomes available Explanatory variables of vaccine hesitancy: female, unmarried, perceiving a lower risk of infection, not being vaccinated against influenza in the past season, not believing in the efficacy of COVID-19 vaccines or not valuing doctor’s recommendations.	Education and communication strategies from heath authorities as well as immunisation programs that remove barriers (e.g., vaccine price and vaccination convenience) seem to be important measures to alleviate public concerns aboutvaccine safety.
(Chen et al., 2021) [68] China *PubMed* ***Hum. Vaccine Immunother.*** **(IF JCR = 4.526; Q1)**	*To understand the willingness and determinants for the acceptance of a COVID-19 vaccine among Chinese* *adults.*	3195 adults Female (63.6%); ≥45 years (13.5%); master’s and above (32.6%); poor health (0.4%); chronic disease, yes (10.4%).	A cross-sectional survey: an online questionnaire Multivariable logistic regression May to June 2020 (before the beginning of the COVID-19 vaccination period).	83.8% were willing to receive a COVID-19 vaccine Explanatory variables of vaccine hesitancy: the risk of vaccines, complacency in health, and low frequency attention to relevant COVID-19 information.	COVID-19 vaccine information (e.g., safety and efficacy issues) should be propagated to ensure vaccination acceptance and coverage.
(Zhao et al, 2021) [69] China Bon ***Vaccines*** **(IF JCR = 4.961; Q1)**	*To assess the willingness of the general population to receive COVID-19 vaccines and identify factors that influence vaccine hesitancy and resistance.*	34,041 respondents Female (53.8%); 18–39 years (60.9%); urban (79.1%); college degree or higher (79.2%); married (77.5%); confirmed or suspected COVID-19 infection (0.3%); quarantine, no (88.6%); chronic diseases (9.1%); normal symptoms of anxiety (78.9%).	Online survey Multinomial logistic regression analyses 29 January 2021 to 26 April 2021 (40.4%); 1–31 March 2021 (51.1%); and 1–26 April 2021 (8.5%) (after the beginning of COVID-19 vaccination).	55.3% of respondents were willing to get vaccinated. Explanatory variables of vaccine hesitancy: female, young and middle-aged, high income, residing in cities and perceived low risk of infection. Vaccine acceptance increased over time, with geographical discrepancies in vaccine hesitancy between provinces.	Tailored public health programs, measures or interventions should consider study findings, such as differences in the profile of vaccine hesitancy between different regions.
**UK**					
(Williams et al., 2020) [70] UK *PubMed* ***Vaccines*** **(IF JCR = 4.961; Q1)**	*To assess key sociodemographic variables and intention to accept a COVID-19 vaccine.*	3436 people (time 1) A total of 2016 people (time 2; 2 months later) Age (≥50 years (45.9% *t*1 and 51.5% t2); female (79.1% *t*1 and 82.1% *t*2); White (96.3 *t*1 and 96.7 *t*2); and <£16,000(9.7% *t*1 and 9.7% *t*2).	Two-wave online survey (prospective study) Logistic regression analyses Time 1 (*t*1): from 20 May to 12 June 2020, weeks 9–12 of national lockdown. Time 2 (*t*2): Two months later (before the beginning of COVID-19 vaccination).	74% respondents reported being willing to receive a COVID-19 vaccine. Explanatory variables of vaccine hesitancy: non-white (i.e., Black, Asian and minority ethnic); low-income levels; low education levels; and no underlying medical conditions/not shielding.	Mass media and social marketing interventions should address the concerns of subpopulations and diverse communities.
(Sherman et al., 2021) [71] UK *PubMed* ***Human Vaccines and Immunotherapeutics*** **(IF JCR 4.526; Q1)**	*To investigate factors associated with the intention to be vaccinated against COVID-19.*	1500 adults Females (51%); White (84.5%); degree equivalent or higher (52.6%); unemployed (37.1%); extremely clinically vulnerable – respondent (29.7%); influenza vaccination last winter, yes (32.3%).	Cross-sectional survey. Linear regression analyses. 14 and 17 July 2020. (before the beginning of COVID-19 vaccination period).	64% respondents declared themselves very likely to become vaccinated against COVID-19. Explanatory variables of vaccine hesitancy: younger age; not being vaccinated against influenza last winter; lower perceived risk of getting COVID-19; a less positive general COVID-19 vaccination beliefs and attitudes; stronger beliefs that the vaccination would cause side effects or be unsafe; lower perceived information sufficiency to make an informed decision about COVID-19 vaccination; an higher endorsement of the notion that only people who are at risk of serious illness should be vaccinated for COVID-19.	Findings should be used to design COVID-19 vaccination campaigns, which could explain the risk of COVID-19 to third parties and necessity for everyone to be vaccinated.
(Sherman et al., 2022) [72] UK Bon ***Public Health*** **(IF JCR = 4.984; Q1)**	*To investigate the factors associated with the intention to get the COVID-19 vaccination following* *initiation of the UK national vaccination programme.*	1500 adults Female (51%); White (84.6%); degree equivalent or higher (54.5%); full-time employment (43.3%); household incomed under GBP 10,000 (6.3%); influenza vaccination last winter (30.5%); extremely clinically vulnerable (22.9%) and extremely clinically vulnerable e other(s) in household (16.9%).	Online cross-sectional survey Linear regression analyses 13–15 January 2021 (after the beginning of COVID-19 vaccination period).	73.5% of participants reported being likely to be vaccinated against COVID-19 Explanatory variables of vaccine hesitancy: not having been/intending to be vaccinated for influenza last winter/this winter; lower beliefs about social acceptability of a COVID-19 vaccine; a lower perceived need for vaccination; adequacy of information about the vaccine; and stronger beliefs that the vaccine is unsafe.	Continued engagement with the public regarding the need COVID-19 vaccination is recommended. For instance, safety issues should be highlighted.
**Australia**					
(Seale et al., 2021) [73] Australia *PubMed* ***BMC Infectious Diseases*** **(IF JCR = 3.667; Q1)**	*To understand the public perceptions regarding a future COVID-19 vaccine in Australia.*	1420 Australian adults (18 years and older) Male (47.7%); less than 50 years (56.6%); not working (41.6%); university degree (38.1%); chronic health condition (25.6%).	A national cross-sectional online survey. Logistic regression model analysis. 18 and 24 March 2020 (before the beginning of COVID-19 vaccination period).	80% (n = 1143) agreed with the statement that getting vaccinated against COVID-19 would be a good way to protect themselves against infection. Explanatory variables of vaccine hesitancy: males, 18–29 year olds, not self-reporting chronic disease, not having private health insurance, and not internationally travelling in 2020.	These findings should support governmental political health measures to identify the appropriate strategies that will support citizens in getting COVID-19 vaccines.
(Alley et al., 2021) [74] Australia *PubMed* ***International Journal of Environmental Research and Public Health*** **(IF JCR = 4.614; Q1)**	*To determine whether willingness to vaccinate changed in the repeated sample and to determine whether willingness* *to vaccinate was associated with demographics, chronic disease or media use.*	2343 Australian adults (both surveys) 55 years old (46%); female (66%), bachelor’s degree or higher (60%); a chronic disease (48%); more than 3 h per day: social media (41%) vs. traditional media (24%).	Two surveys A generalized linear mixed model (first objective) and a multinominal logistic regression (second objective) April and August 2020 (before the beginning of COVID-19 vaccination period).	Willingness to be vaccinated: April (87%) and August (85%). Explanatory variables of vaccine hesitancy: people with a certificate or diploma; infrequent users of traditional media; women.	Strategies to promote COVID-19 vaccination should target women, and people with a certificate or diploma, via non-traditional media channels.
(Attwell et al., 2021) [75] Australia *PubMed* ***PloS One*** **(IF JCR = 3.752; Q1)**	*To identify factors that differentiate those who are undecided from those who are either willing or unwilling to accept a prospective COVID-19 vaccine.*	1313 adults Female (60%); mean age; 58 (SD = 13.20); parents with children living at home (31%).	Online survey Multinomial logistical regression May 2020 (before the beginning of COVID-19 vaccination period).	65% were willing to vaccinate, with 27% being in the “maybe” category. Explanatory variables of vaccine hesitancy (respondents were more likely to be in the COVID-19 vaccine “maybe” than ”yes” group): perceived COVID-19 to be less severe; had less trust in science; were less willing to get a flu vaccine and were female. Explanatory variables of vaccine hesitancy (respondents were more likely to be in the COVID-19 vaccine “no” than “maybe” group): they did perceive COVID-19 to be a hoax	The dimension of the undecided group about receiving a COVID-19 vaccine (over a quarter (27%)) suggests that this group will be important to the effective nationwide rollout of the vaccine. Research to understand the motivation of undecides is urgently needed.
**Asia**					
(Albahri et al., 2021) [76] United Arab Emirates Bon ***Frontiers in Public Health*** **(IF JCR = 6.461; Q1)**	*To evaluate the current vaccine hesitancy in a segment of the United Arab Emirates (UAE) general public and its associated factors.*	2705 respondents Female (72.5%); 44 or less years (74.2%); Emirati (69.8%); postgraduate (21.5%); employed (58.3%); more than two adults in the household (82.3%); at least one child in the household (80.1%); infected with COVID-19 (3.5%); or flu vaccine in the last 2 years, yes (26.9%).	Online cross-sectionalMultivariable logistic regression analysis 14 to 19 September 2020 (before the beginning of COVID-19 vaccination period).	60.1% respondents were willing to get the COVID-19 vaccine. Explanatory variables of vaccine hesitancy: female, Emiratis, older participants, retired, unemployed, postgraduate, not receiving the influenza vaccine within the past 2 years, not believing in the seriousness of the COVID-19 situation or the vaccine’s ability to control the pandemic, perceiving the effect of the disease to have a lower effect on one’s personal health, not believing that the public authorities are handling the pandemic adequately. Vaccine safety, side effects and the belief that one needs to develop immunity naturally were the top reasons for vaccination hesitancy.	Initiatives are recommended to fight vaccine misinformation. For instance, providing information on vaccine safety and efficacy (e.g., side effects or highlighting the advantages of getting herd immunity through vaccination over natural acquired immunity).
(Alremeithi et al., 2021) [77] United Arab Emirates Bon ***Frontiers in Public Health*** **(IF JCR = 6.461; Q1)**	*To assess* *The knowledge, attitude and practices toward infection* *by SARS-CoV-2, including vaccine acceptance. ***	1882 people Females (80.7%), younger than 40 (67.7%), males (19.3%), UAE nationals (78.2%), healthcare worker (9.9%), held a bachelor’s degree or above (54.5%), healthy (73.3%).	Questionnaire Multivariate linear regression 4–14 April 2020 over a period of 10 days. (Before the beginning of COVID-19 vaccination period.)	89% respondents agreed that SARS-CoV-2 infection would be successfully controlled. Explanatory variables of vaccine hesitancy: less likely to wear masks, older age, UAE nationals, lower knowledge scores on SARS-CoV-2 infection and females.	Intensification of awareness programs and good practices were recommended.
(Harapan et al., 2020) [78] Indonesia *PubMed* ***Frontiers in Public Health*** **(IF JCR = 6.461; Q1)**	*To assess the acceptance of a 50- or 95%-effective COVID-19 vaccine, when it becomes available in southeast Asia, among the general population in Indonesia.*	1359 respondents Female (65.7%); less than 50 years (94.8%); university graduate/postgraduate (66.1%); single (55.9%).	A cross-sectional online survey A logistic regression model. 25 March and 6 April 6 2020 (before the beginning of the COVID-19 vaccination period).	93.3% of respondents would like to be vaccinated (95% effectiveness) and 67.0% of respondents would like to be vaccinated (50% effectiveness). Explanatory variables of vaccine hesitancy (first scenario, 95% COVID-19 vaccine effectiveness): not being a healthcare professional, and lower perceived risk of getting infected were associated. Explanatory variables of vaccine hesitancy (second scenario, 50% COVID-19 vaccine effectiveness): being a healthcare professional was the only characteristic associated with vaccine acceptance.	Acceptance of the COVID-19 vaccine was influenced by their effectiveness in the present study. It seems governments need to address the perceived risk in communities and more vaccination strategies/measures for lower effective vaccines.
(Al-Mohaithef and Padhi, 2020) [79] Saudi Arabia *PubMed* ***Journal of Multidisciplinary Healthcare*** **(IF JCR = 2.919; Q1)**	*To assess the prevalence of the acceptance of COVID-19 vaccine and their determinants* *among people in Saudi Arabia.*	992 respondents Female (65.83%); above 45 years (5%); married (51.61%); Saudi (82.06%); graduate (50.1%); unemployed (39.72%).	Questionnaires (social media platforms and email) Logistic regression analysis. Timeframe was not detailed.	64.7% showed interest in accepting the COVID-19 vaccine. Explanatory variables of vaccine hesitancy: people with less than 45 years; non-married; lower perceived risk of being infected; less trust in the health system.	Addressing sociodemographic determinants as well as tailored health education interventions to promote COVID-19 vaccination,
(El-Elimat et al., 2021) [80] Jordan Bon ***PLoS One*** **(IF JCR = 3.752; Q1)**	*To investigate* *the acceptability of COVID-19 vaccines and its predictors in addition to the attitudes towards* *these vaccines among the public in Jordan.*	3100 respondents Females (67.4%); >35 years (34.3%); married (49.8%); with children (46.1%); postgraduate (21.4%), health-related area (53.8%); employed (46.4%); insured (78.1%); smoker (21.9%); chronic disease (13.4%); influenza vaccine (9.4%); or infected with COVID-19 (37.1%).	Online, cross-sectional, and self-administered questionnaire Logistic regression analysis November 2020 (before the beginning of vaccination period).	37.4% declared that they would accept COVID-19 vaccines Explanatory variables of vaccine hesitancy: females, not receiving the seasonal influenza vaccine, believing that vaccines are not safe, not being available to pay these vaccines, above 35 years old, employed, believing that there was a conspiracy behind COVID-19, not trust in any source of information on COVID-19 vaccines	Study finding should be considered in informative vaccination campaigns, such as to offer transparent information on the safety and efficacy of vaccines (e.g., production technology).
(Green et al., 2021) [81] Israel Bon ***Israel Journal of Health Policy Research*** **(IF JCR = 2.465; Q1)**	*To assess ethnic and sociodemographic factors in Israel associated with attitudes towards COVID-19 vaccines prior to their introduction.*	957 adults 55% Females; Jews (63%); Arabs (37%); single (22.6%); academic degree (58.8%); religious (20.4%).	Cross-sectional survey During October 2020 (before the beginning of vaccination period).	People who want to be vaccinated immediately: men (27.3% of the Jewish and 23.1% of the Arab respondents) and women (13.6% of Jewish and 12.0% of Arab respondents). Explanatory variables of vaccine hesitancy: lower level of education, the belief that the government restrictions were too lenient, the frequency of socialising prior to the pandemic (goes out frequently), Arabs and being female.	Besides ensuring an effective communication to the general population, vaccine promotion campaigns should be designed to effectively communicate to target groups.
(Khaled et al., 2021) [82] Qatar Bon ***Vaccines*** **(IF JCR = 4.961; Q1)**	*To estimate* *the prevalence and identify potential determinants of vaccine willingness: acceptance (strongly* *agree), resistance (strongly disagree) and hesitance (somewhat agree, neutral, somewhat disagree).*	1038 respondents Female (31.7%); 40+ years (42.4%); married (73.3%); employed (76.8%); Arab (55.8%); lives with others (83.1%); chronic disease, Yes (25.8%); COVID-19 infection (8.2%); quarantine status, yes (22.1%); very concerned with COVID-19 infection (32.9%).	Phone survey Bivariate and multinomial logistic regression modelsDecember 2020 and January 2021 (before and after the beginning of the COVID-19 vaccination period).	42.7% of respondents declared that they were willing to be vaccinated. Explanatory variables of vaccine hesitancy: female gender, Arab ethnicity, migrant status/type and vaccine side effects.	Study findings should be used to define tailored public health programs, measure, or interventions.
(Qamar et al., 2021) [83] Pakistan Bon ***Cureus*** **(no impact factor; paper also available in PubMed)**	*To assess the acceptance of the* *COVID-19 vaccine among the general public in Pakistan.*	936 respondents Female (59.6%); 18–19 years (38.4%); bachelor’s or master’s degree (84.8%); unemployed (15.9%); <10,000 (in Pakistani rupees) (5.6%); married (22.3%); healthcare workers or medical students (46%); presence of COVID-19 infection in family or friends (45.6%); comorbidities (13.9%).	Questionnaire Logistic regression analysis January 2021 to February 2021 (before the beginning of COVID-19 vaccination period).	70% agreed to be vaccinated if recommended. Explanatory variables of vaccine hesitancy: female gender, not being a healthcare worker, not being a medical student, no COVID-19 infection in family or friends, not trusting in the reliability of media sources regarding COVID-19, not trusting in the national government to control the pandemic and not believing that COVID-19 can be debilitating and dangerous to health.	These finding should be used to define information interventions. For instance, disseminating credible information through healthcare workers. Government officials, social media influencer channels or media outlets.
(Wong et al., 2021) [84] Hong Kong *PubMed* ***Vaccine*** **(IF JCR = 4.169; Q1)**	*To examine the factors associated with acceptance of vaccine based on* *(1). constructs of the Health Belief Model (HBM), (2). trust in the healthcare system, new vaccine platforms* *and manufacturers and (3). self-reported health outcomes.*	1200 respondents (adults) Female (71.3%); ≥65 years (46.8%); married (77.5%); education: tertiary or above (22.9%); unemployed (3%); chronic conditions (51.4%).	Random telephone survey Multivariate logistic regression 27 July 2020 to 27 August 2020 (before the beginning of the COVID-19 vaccination period).	42.2% of respondents indicated acceptance of COVID-19 vaccine. Explanatory variables of vaccine hesitancy: lower perceived severity (severity and consequences of having COVID-19 by 4 items); lower perceived benefits of COVID-19 vaccine (value or efficacy of receiving COVID-19 vaccine by 4 items); lower cues to action (triggers for receiving COVD-19 vaccination by 4 items, including recommendations by the government, physicians, family members and friends, respectively); lower self-reported negative health outcomes (measured by the presence of chronic conditions and the self-report health-related quality of life (HRQoL)) and lower trust in healthcare system or vaccine manufacturers.	Study findings are relevant formulation and implementation of vaccination strategies.
(Hanna et al., 2022) [85] Lebanon Bon ***Journal of Pharmaceutical Policy and Practice*** **(no IF JCR; Q1; indexed in Scopus)**	*To assess the COVID-19 vaccines’ acceptance and related determinants in the Lebanese population.*	1209 respondents Females (67.1%); living in rural area (25.9%); single (41.4%); living in the same household with children (44.8%); advanced degree (38.9%); employed (74.7%); obese (14.2%); allergies to medication (11.7%); no previous COVID-19 infection (79.4%).	Online questionnaire via social media platforms Binary logistic regression 16 February through 25 February 2021 (Before the beginning of COVID-19 vaccination period).	63.4% reported they would accept COVID-19 vaccination. Explanatory variables of vaccine hesitancy: lower general vaccine knowledge index, living in a rural residential area, not having hypertension, having a food allergy, lower fear of experiencing COVID-19 infection and not receiving or not wanting to receive influenza vaccine.	Education and awareness programs are needed to improve knowledge about COVID-19 infection and vaccination, such as among residents of rural areas.
(Hwang et al., 2022) [86] South Korea Bon ***Human Vaccines and Immunotherapeutics*** **(IF JCR 4.526; Q1)**	*To investigate (1) the prevalence and reasons for COVID-19 vaccine* *hesitancy, (2) subgroups that had higher rates of vaccine hesitancy and (3) vaccine hesitancy predictors.*	13,021 adults Female (50.2%); 20–39 years (33.5%); spouse (71.5%); college graduate (57.9%); employed (67.3%); good health status (94.6%); or no religion (71.5%).	National survey Logistic multivariate regression October to December 2020 (before the beginning of COVID-19 vaccination period).	60.2% of the participants were not vaccine-hesitant. Explanatory variables of vaccine hesitancy: lack of confidence in the COVID-19 vaccine, less or no fear of COVID-19, unstable job status, decreased/lower family income, experienced worsening health status during pandemic, younger age, no religious affiliation and political conservatism	Identified predictors variables should be considered in future studies.
**European Union**					
(Prati et al., 2020) [87] Italy *PubMed* ***Health Education Research*** **(IF JCR = 2.221; Q2)**	*To determine* *the extent to which Italian people intend to* *receive a vaccine against SARS-CoV-2 and to investigate* *its associations with worry, institutional* *trust and beliefs about the non-natural origin* *of the virus.*	624 adults Women (54%); aged between 18 and 72 years (average = 32.31; SD = 12.69; employed (52.4%); ethnic minority group (4.0%).	Questionnaire: online platform. Multinomial logistic regression Model. April 2020. (Before the beginning of COVID-19 vaccination).	75.8% respondents intended to receive a vaccine. Explanatory variables of vaccine hesitancy: a lower level of worry to health risks; a lower level of institutional trust in Italian government, Ministry of Health and physicians and declaring to not know about the non-natural origin of the virus.	Trust, conspiracy beliefs and worry should be considered when designing new vaccination programs.
(Bagić et al., 2022) [88] Croatia Bon ***Croatian Medical Journal*** **(IF JCR = 2.415; Q3)**	*To assess the determinants and reasons for coronavirus* *disease 2019 (COVID-19) vaccine hesitancy in Croatia.*	758 respondents Female (58.2%); 45 or more years (62.8%); bachelor’s, master’s degree or higher (45.4%); settlement size up to 10000 (46.4%); children in the household (0–17) (34.1%); Trust in the government (40.9%); or assessment of SARS-CoV-2 virus infectionrisk—small or no risk (23.3%).	A sociological surveyBinary logistic regression 4 March and 11 April 2021 (after the beginning of COVID-19 vaccination period).	63.9% declared they would receive a COVID-19 vaccination. Explanatory variables of vaccine hesitancy: women, younger age groups (especially 25–34 year olds), persons residing in households with children, smaller settlements, persons with lower levels of education, perceiving infection as a low risk or low levels of trust in the five main actors responding to the COVID-19 pandemic (the National Civil Protection Headquarters, government, healthcare system, scientists-researchers and media).	Study finding should be considered when designing informative vaccination campaigns.
**Latin America**					
(Carnalla et al., 2021) [89] Mexico Bon ***Salud Publica de Mexico*** **(IF JCR 2.259; Q3)**	*To estimate the willingness to vaccinate against* *COVID-19 (acceptance) in the Mexican population and to identify* *socioeconomic factors associated with vaccine hesitancy* *and refusal.*	10,796 respondents Female (54.5%); 40 or more years (46.1%); elementary school (30.8%); low socioeconomic level (33.3%); unemployed (29.6%); chronic disease, yes (19.9%).	Data from the COVID-19 National Health and Nutrition Survey Multinomial logistic regression August to November 2020 (before the beginning of COVID-19 vaccination period).	62.3% declared they would accept COVID-19 vaccination Explanatory variables of vaccine hesitancy: female, older age, lower educational level, lower socioeconomic status and working in the informal sector.	National campaigns on COVID-19 vaccination should target the more vaccine-hesitant subgroups.
(Moore et al., 2021) [90] Brazil Bon ***Vaccine*** **(IF JCR = 4.169; Q1)**	*To know the vaccine hesitancy profile in Brazil.*	173,178 respondents Females (67.1%); ≥40 years (70.6%); White (71%); completed secondary or more (81.8%); ≤USD 197.17 (5.9%); children, yes (64.2%).	Anonymous online survey Multivariate logistic model 22 to 29 January 2021 (after the beginning of COVID-19 vaccination period).	10.5% of respondents were vaccine-hesitant. Explanatory variables of vaccine hesitancy: assigning importance to the vaccine´s efficacy, fear of adverse reactions, assigning importance to the vaccine´s country of origin, male gender, having children, 9 years of schooling or less, living in the Central-West region, age ≥ 40 years, monthly income <USD 788.68., White, not residing in state capital, less fear of catching COVID-19, no family member died or admitted to ICU for COVID-19.	The identified explanatory variables can facilitate the elaboration of communication strategies to increase vaccine adherence, although the global vaccine hesitancy was low.
**Caribbean**					
(De Freitas et al., 2021) [91] Trinidad and Tobago Bon ***Lancet Regional Health Americas*** **(without if JCR, launched July 2021, indexed in PubMed)**	*To evaluate public trust in information sources, confidence in institutions and COVID-19 vaccine willingness in Trinidad and Tobago.*	615 respondents Female (66%); 18–29 years (53.8%); university or higher education (84.6%); healthcare professional (31.7%); chronic illness, yes (11.7%).	Online survey Binomial logistic regression 10 November to 7 December 2020 (after the beginning of COVID-19 vaccination period).	62.8 % of participants declared that they would get the COVID-19 vaccine. Explanatory variables of vaccine hesitancy: not trusting in medical sectors to manage COVID-19, not believing that everyone should be vaccinated according to the national immunisation schedule and not willing to get flu vaccines.	Study findings may be used to design and prioritise future intervention areas.
**Africa**					
(Kollamparambil et al., 2021) [92] South Africa Bon ***BMC Public Health*** **(IF JCR = 4.169; Q1)**	*To assess the level of COVID19 vaccine hesitancy in* *South Africa, identify the socioeconomic patterns in vaccine hesitancy and highlight insights from the national* *survey that can inform the development of a COVID-19 vaccination acceptance communication campaign.*	4440 respondents Age, years (mean = 40.47); minimum = 0; maximum = 1: chronic illness (mean = 0.168); COVID-19 awareness (mean = 0.096); male (mean = 0.482); married/with partner (mean = 0.479); urban (mean = 0.76).	Nationally representative National Income Dynamics Study—Coronavirus Rapid Mobile Survey (NIDS-CRAM) survey Logistic regression models 2–28 February 2021 and March 2021 (before and after the beginning of COVID-19 vaccination period).	55% of the population had a strong acceptance of the vaccine. Explanatory variables of vaccine hesitancy: lower perceived risk of infection, lower perceived efficacy of vaccine, lower awareness of COVID19-related information, lower income, non-black African population group (denial non-black African, religious), the young, less educated and those without partners.	Clearer information on the risk messaging on COVID-19 vs. efficacy and safety of the vaccines. Information campaigns should target the identified groups.

* Only some of the collected sociodemographic variables are reported here due to space limitations; ** studies that not exclusively evaluate predictors of vaccine acceptance and/or hesitancy.
